# Supplementary material for: Modelling the impact of vaccine hesitancy in prolonging the need for Non-Pharmaceutical Interventions to control the COVID-19 pandemic
Source: Commun Med (Lond). 2022 Feb 10;2:14. doi: 10.1038/s43856-022-00075-x (PMC9053271; doi:10.1038/s43856-022-00075-x)
Supplement: Supplementary file 2 — Supplementary Materials [file 43856_2022_75_MOESM2_ESM.pdf]

Modelling the impact of vaccine hesitancy in prolonging the need for Non-Pharmaceutical Interventions to control the COVID-19 pandemic

Supplementary Material

Daniela Olivera Mesa *[1], Research Postgraduate Student, d.olivera-mesa17@imperial.ac.uk

Alexandra B Hogan[1], Research Fellow.

Oliver J Watson[1], Research Associate.

Giovanni D Charles[1], Research Software Engineer.

Katharina Hauck[1], Reader in Health Economics.

Azra C Ghani[1], Professor in Infectious Disease Epidemiology.

Peter Winskill[1], Research Fellow.

1. MRC Centre for Global Infectious Disease Analysis; and the Abdul Latif Jameel Institute for Disease and Emergency Analytics (J-IDEA), School of Public Health, Imperial College London, London, United Kingdom

1 Supplementary Methods

1.1 Mathematical model (Modified from Hogan et al. [1])

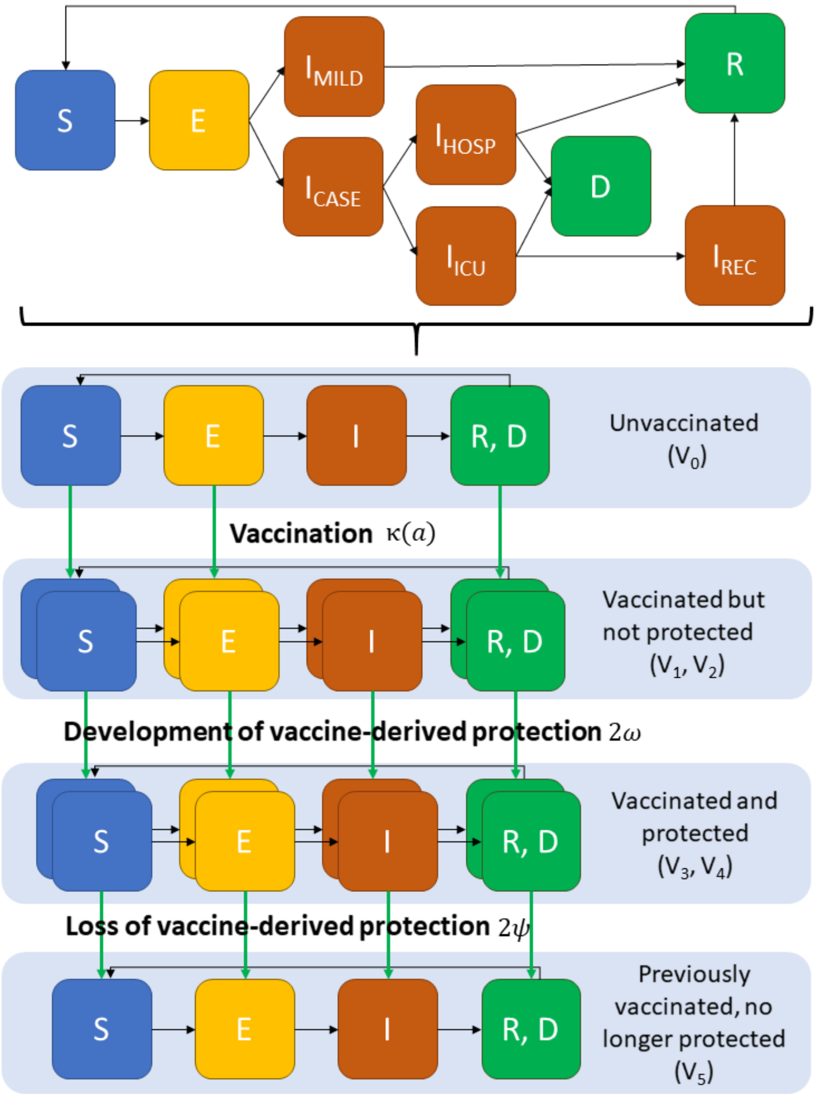

***Figure S1: Schematic of the SARS-CoV-2 transmission model**. The schematic Susceptible-Exposed- Infected-Recovered (SEIR) model representation is based on the model described by Hogan et al Hogan et al. [1]Infected individuals are disaggregated in mild cases and the disease clinical cases, which includes hospitalisation and intensive care. Vaccination status is included as a new dimension in the model. Only individuals in the susceptible (S), exposed (E) and recovered (R)* compartments *can be vaccinated*.

1.2 Description

A previously published mathematical model for SARS-CoV-2 transmission was used to predict the public health impact of vaccine hesitancy. The model captures the disease dynamics among nine different infection states, stratified by age and vaccination status (Figure S1).

- S = uninfected and therefore **susceptible** to infection
- E = **exposed** to infection but not yet infectious
- I_{MILD} = **infected** and infectious with mild infection that does not require hospitalisation (this includes both symptomatic and asymptomatic infection)
- I_{CASE} = **infected** and infectious with disease that will require hospitalisation
- I_{HOSP} = cases that have been **hospitalised** in a general ward bed
- I_{ICU} = cases that have been admitted to **an intensive care unit (ICU)**
- I_{REC} = cases that have been stepped down from ICU into a general ward bed for **recovery**
- D = cases that have **died**
- R = infections and cases that have **recovered** and are immune to re-infection

Disease states E, I_{CASE}, $I_{HOSPITAL}$, I_{ICU} and R are split into two sequential states such that the durations of stay are Erlang-distributed. Individuals become infected at a rate that depends on the number of people in states I_{MILD} and I_{CASE} and the transmission probability. Following infection, individuals in the I_{CASE} state proceed to an ICU unit (I_{ICU}) or hospitalisation in general ward (I_{HOSP}) at rates based on age specific probabilities and country-specific hospital bed capacity. After infection, cases either died (D) or recover (R) following the path shown in Figure S1 (with the final outcome tracked by splitting both the I_{HOSP} and I_{ICU} states into two compartments reflecting those that die or recover respectively in order to capture the different durations of stay associated with these outcomes). Finally, recovered individuals can lose naturally acquired immunity and then return to the susceptible state. Additional constraints are included in the hospitalisation pathway to capture situations in which the need exceeds capacity; with those that do not receive appropriate care experiencing higher death rates[2]. These constrains are country-dependent for the country-specific scenarios and are described in Table S3.

The level of transmission in the model is parameterised by the reproduction number, R_t, in the absence of vaccine or naturally induced immunity. This is equal to R_0 at the start of the simulation and may be modified forwards in time by the introduction of non-pharmaceutical interventions (NPIs). The transmission probability is obtained as the ratio between the reproductive number and the leading eigen value of the next generation matrix, which depends on duration of infectiousness, the age-stratified mixing matrix and age-dependent probability of hospitalisation.

To model vaccination, the population is stratified into 6 vaccination classes, with the vaccinated states split into two compartments (v1/v2 and v3/v4) to generate Erlang-distributed waiting times:

- v_0 = unvaccinated
- v_1 and v_2 = vaccinated but not yet protected, reflecting the two-dose vaccine schedule and need to wait approximately 28 days from dose 1 for protection to develop
- v_3 and v_4 = vaccinated and protected
- v_5 = previously vaccinated but no longer protected, used to capture waning vaccine efficacy.

Only individuals who are susceptible, in the latent period, or recovered can be vaccinated. It is assumed that individuals receive a single two-dose schedule (with no drop-out) and that vaccine efficacy is generated after the second dose. This approximation therefore ignores the partial efficacy obtained from the first dose. We assume that the vaccine protects against infection – by reducing the transmission parameter by a constant factor; and against severe disease in breakthrough infections – by reducing the rate of hospitalisation by a constant factor.

Vaccines are distributed by age groups at a constant rate and a matrix of coverage targets that represents prioritisation strategies. In this matrix rows represent ordered prioritisation steps and columns the age group. The target coverage per age group was changed according to the different scenarios modelled (Table S5). In each prioritisation step (s), vaccines are given at rat to all age groups that satisfy. Once all target coverages are met in the current prioritisation step, the step is incremented, and the process repeated. When all coverage targets in the final prioritisation step are met, vaccination is ceased. This therefore means that if vaccine uptake is lower, vaccines will be distributed to younger age-groups rather than waiting for a set period of time for each age-group. This is consistent with a constant supply of vaccines and best matches the roll-out of vaccines to date in high-income countries.

The parameters for the model are shown in Section 1.2 and the full equations (reproduced from Supplementary Material in Hogan et al. [1]) are shown in Section 1.3.

1.3 Parameters

Table S1: Epidemiological parameter descriptions and values: Modified from Hogan et al[1].

Parameter	Symbol	Value	Description
Transmission parameter	β	-	Calculated from R_t as described by Walker et al[2]
Basic reproduction number	R_0	3.0<br>3.91 (France)<br>4.22 (Germany)<br>4.16 (U.K)	For the representative scenario, value was estimated from European data consistent with a doubling time of 3.5 days[2]. Country values were obtained by fitting to the reported deaths.
Mean Latent Period	$\frac{1}{\alpha}$	4.6 days	Estimated at 5.1 day[3-5]. The last 0.5 days are incorporated in the infectious periods to capture pre-symptomatic infectivity
Mean Duration of Mild Infection	$\frac{1}{\gamma_1}$	2.1 days	Incorporates 0.5 days of infectiousness prior to symptoms. In combination with mean duration of severe illness this gives a mean serial interval of 6.75 days[6].
Mean Duration of Severe Infection Prior to Hospitalisation	$\frac{1}{\gamma_2}$	4.5 days	Mean onset-to-admission of 4 days[7]. Values in the wider literature range from 1.2 days to 12 days[3-5,8,9]. Includes 0.5 days of infectiousness prior to symptom onset.
Mean Duration of Hospitalisation for non-critical cases if survive	$\frac{1}{\gamma_{3,1}}$	9 days	Median of values identified in [8-12]
Mean Duration of Hospitalisation for non-critical cases if die	$\frac{1}{\gamma_{3,0}}$	9 days	Median of values identified in [8-12]
Mean Duration in ICU if survive	$\frac{1}{\gamma_{4,1}}$	14.8 days	Mean duration in ICU of 13.3 days from a study across 42 countries[13]. Ratio of duration in critical care if die: duration in critical care if survive of 0.75 and 60.1% probability of survival in ICU[14].
Mean Duration in ICU if die	$\frac{1}{\gamma_{4,0}}$	11.1 days	Mean duration in ICU of 13.3 days from a study across 42 countries[13]. Ratio of duration in critical care if die: duration in critical care if survive of 0.75 and 60.1% probability of survival in ICU[14].
Mean Duration in Recovery after ICU	$\frac{1}{\gamma_5}$	3.4 days	Working assumption
Mean duration of naturally acquired immunity	$1/\rho$	365 days	Assumed value based on published data of protection to reinfection. Protection is reported to last at least 8 months [15-17]
Infection fatality ratio (IFR)	$\mu(a)$	See Table S4	Age-dependent[18]
Proportion of infections hospitalised	$\phi_1(a)$	See Table S4	Age-dependent[19].
Proportion of hospitalisations requiring ICU	$\phi_2(a)$	See Table S4	Age-dependent[19].

Table S2: Vaccination parameter descriptions and values

Parameter	Symbol	Value	Description
Vaccine efficacy against infection	$1 - v_{inf}(a)$	94%; 63%	We assumed infection-blocking efficacy is the same as reported vaccine efficacy against clinical disease. Values were selected to cover the range of approved vaccines efficacies reported to date [20,21]
Vaccine efficacy against disease	$1 - v_{dis}(a)$	60%	Estimate based on reported vaccine effectiveness data in the UK which suggests ~86% efficacy against hospitalisation/death compared to ~65% against mild disease for a single dose of the Pfizer vaccine [22,23]. The assumed value of 60% generates 98% efficacy against hospitalisation/death for the high efficacy vaccines and 85% for the moderate efficacy vaccine, with both representing two dose schedules.
Vaccine mean duration of protection	$1/\psi$	5000 days	Assumption generating durable immunity for 1 year simulations.
Rate of vaccination	$\kappa(a)$	135 399 per day (representative) 183 834 per day (U.K) 176 810 per day (France) 237 142 per day (Germany)	Population-dependent: set such that number of people vaccinated per day achieves vaccination of all individuals aged 15 years and above in a 10-month period. Representative scenario assumes a total population of 50 million individuals and U.K age demographics
Mean time to develop vaccine-acquired immunity following second dose	$1/\psi$	7 days	Based on immunogenicity data from Phase II trials in which antibody titres plateau ~7 days post dose 2 [24-28]
Vaccine schedule	-	21 days	2 doses modelled 21 days apart[20,21,29,30]. Efficacy follows 2nd dose (so only modelling final dose of any vaccine schedule)

Table S3: Hospital capacity parameters per country

Parameter	Country	Value	Description
Maximum hospital beds per capita	U.K	4.63	Values taken from R package squire using methods described by Walker et al. [2]For the representative scenario U.K values were implemented
	France	6.5	
	Germany	7.4	
Maximum ICU beds per capita	U.K	0.15	Values taken from R package squire using methods described by Walker et al. [2] For the representative scenario U.K values were implemented
	France	0.21	
	Germany	0.24	

Table S4: Age-dependent parameters for hospitalisation and death. *Reproduced from Hogan et al[1].*

Age group (years)	Proportion of infections hospitalised[19]	Proportion of hospitalised cases requiring ICU[19]	Proportion of hospital deaths occurring in ICU	Proportion of non-ICU cases dying	Proportion of ICU cases dying	Infection fatality ratio (IFR)[18]
0 to 4	0.001	0.181	0.8	0.013	0.227	0.00004
5 to 9	0.001	0.181	0.8	0.014	0.252	0.00007
10 to 14	0.002	0.181	0.8	0.016	0.281	0.00011
15 to 19	0.002	0.137	0.8	0.016	0.413	0.00017
20 to 24	0.003	0.122	0.8	0.018	0.518	0.00026
25 to 29	0.005	0.123	0.8	0.020	0.573	0.00041
30 to 34	0.007	0.136	0.8	0.023	0.576	0.00064
35 to 39	0.009	0.161	0.8	0.026	0.543	0.00100
40 to 44	0.013	0.197	0.8	0.030	0.494	0.00156
45 to 49	0.018	0.242	0.8	0.036	0.447	0.00245
50 to 54	0.025	0.289	0.8	0.042	0.417	0.00384
55 to 59	0.036	0.327	0.8	0.050	0.411	0.00601
60 to 64	0.050	0.337	0.8	0.056	0.443	0.00941
65 to 69	0.071	0.309	0.8	0.060	0.539	0.01473
70 to 74	0.100	0.244	0.6	0.123	0.570	0.02307
75 to 79	0.140	0.160	0.4	0.184	0.643	0.03613
80+	0.233	0.057	0.15	0.341	0.993	-*
80 to 84	-	-	-	-	-	0.05659
85 to 89	-	-	-	-	-	0.08862
90+	-	-	-	-	-	0.17370

* To standardise input age groups for modelling, IFRs in 80 to 84, 85 to 89 and 90+ years age groups are aggregated to the 80+ years age group using country-specific demography.

Table S5 : Median coverage per age group for vaccine hesitancy scenarios, *10% and 90% quantiles are shown in parenthesis.*

Survey results age group	Simulations' age group	France	Germany	U.K.	Representative scenario (Europe values)
	0-4	0	0	0	0
	5-9	0	0	0	0
	10-14	0	0	0	0
18-24	15-19	0.47 (0.41-0.52)	0.61(0.54-0.66)	0.69(0.64-0.75)	0.58(0.56-0.60)
	20-24	0.47 (0.41-0.52)	0.61(0.54-0.66)	0.69(0.64-0.75)	0.58(0.56-0.60)
25-24	25-29	0.36 (0.32-0.41)	0.57(0.52-0.61)	0.76(0.71-0.80)	0.57(0.56-0.59)
	30-34	0.36 (0.32-0.41)	0.57(0.52-0.61)	0.76(0.71-0.80)	0.57(0.56-0.59)
35-44	35-39	0.35 (0.31-0.39)	0.63(0.58-0.68)	0.72(0.68-0.76)	0.60(0.58-0.62)
	40-44	0.35 (0.31-0.39)	0.63(0.58-0.68)	0.72(0.68-0.76)	0.60(0.58-0.62)
45-54	45-49	0.51(0.46-0.55)	0.57(0.53-0.62)	0.76(0.71-0.80)	0.65(0.63-0.66)
	50-54	0.51(0.46-0.55)	0.57(0.53-0.62)	0.76(0.71-0.80)	0.65(0.63-0.66)
55-64	55-59	0.49 (0.45-0.54)	0.63(0.59-0.67)	0.86(0.82-0.89)	0.69 (0.68-0.71)
	60-64	0.49 (0.45-0.54)	0.63(0.59-0.67)	0.86(0.82-0.89)	0.69 (0.68-0.71)
65+	65-69	0.67(0.62-0.72)	0.78 (0.74-0.81)	0.91(0.87-0.93)	0.80 (0.79-0.82)
	70-74	0.67(0.62-0.72)	0.78 (0.74-0.81)	0.91(0.87-0.93)	0.80 (0.79-0.82)
	75-79	0.67(0.62-0.72)	0.78 (0.74-0.81)	0.91(0.87-0.93)	0.80 (0.79-0.82)
	80+	0.67(0.62-0.72)	0.78 (0.74-0.81)	0.91(0.87-0.93)	0.80 (0.79-0.82)

***Table S6: Population demographics**. Data on the population size and age distribution of the country was taken from https://population.un.org/wpp/*

Age	France	Germany	U.K.	Representative scenario
0-4	3 619 987	4 058 679	3 924 490	2 890 500
5-9	3 907 487	3 822 225	4 119 566	3 034 179
10-14	3 995 688	3 811 645	3 956 340	2 913 959
15-19	3 887 789	4 118 971	3 686 133	2 714 943
20-24	3 697 019	4 553 436	4 074 640	3 001 090
25-29	3 674 010	4 823 925	4 484 067	3 302 645
30-34	3 941 533	5 441 865	4 706 828	3 466 715
35-39	4 069 517	5 430 155	4 588 196	3 379 339
40-44	3 943 397	5 059 667	4 308 130	3 173 062
45-49	4 382 270	5 183 684	4 296 121	3 164 217
50-54	4 363 401	6 681 221	4 634 540	3 413 472
55-59	4 271 656	6 807 172	4 538 925	3 343 049
60-64	3 973 248	5 820 703	3 905 016	2 876 157
65-69	3 791 538	4 823 439	3 381 761	2 490 765
70-74	3 523 616	3 833 823	3 388 488	2 495 719
75-79	2 204 088	3 637 591	2 442 147	1 798 712
80+	4 027 268	5 875 744	3 450 616	2 541 478

1.4 Mathematical model equations

1.4.1 Vaccination group v_0 - unvaccinated

$$\frac{dS(t,a,v_0)}{dt} = 2\rho R_2(t,a,v_0) - \beta\frac{S(t,a,v_0)}{N}\sum_{a'} c(a,a')[\sum_{v}(I_{MILD}(t,a',v) + I_{CASE}(t,a',v))] - \kappa(a)S(t,a,v_0)$$

$$\frac{dE_1(t,a,v_0)}{dt} = \beta\frac{S(t,a,v_0)}{N}\sum_{a'} c(a,a')[\sum_{v}(I_{MILD}(t,a',v) + I_{CASE}(t,a',v))] - 2\alpha E_1(t,a,v_0) - \kappa(a)E_1(t,a,v_0)$$

$$\frac{dE_2(t,a,v_0)}{dt} = 2\alpha E_1(t,a,v_0) - 2\alpha E_2(t,a,v_0) - \kappa(a)E_2(t,a,v_0)$$

$$\frac{dI_{MILD}(t,a,v_0)}{dt} = (1-\phi_1(a))(2\alpha E_2(t,a,v_0)) - \gamma_1 I_{MILD}(t,a,v_0)$$

$$\frac{dI_{CASE,0}(t,a,v_0)}{dt} = \phi_1(a)(2\alpha E_2(t,a,v_0)) - 2\gamma_2 I_{CASE,0}(t,a,v_0)$$

$$\frac{dI_{CASE,1}(t,a,v_0)}{dt} = 2\gamma_2 I_{CASE,0}(t,a,v_0) - 2\gamma_2 I_{CASE,1}(t,a,v_0)$$

$$\frac{dI_{HOSPITAL,0}(t,a,v_0,0{,}0)}{dt} = (1-\delta(H))\mu(a)(1-\phi_2(a))2\gamma_2 I_{CASE,1}(t,a,v_0) - 2\gamma_{3,0} I_{HOSPITAL,0}(t,a,v_0,0{,}0)$$

$$\frac{dI_{HOSPITAL,1}(t,a,v_0,0{,}0)}{dt} = 2\gamma_{3,0} I_{HOSPITAL,0}(t,a,v_0,0{,}0) - 2\gamma_{3,0} I_{HOSPITAL,1}(t,a,v_0,0{,}0)$$

$$\frac{dI_{HOSPITAL,0}(t,a,v_0,1{,}0)}{dt} = \delta(H)\mu(a)(1-\phi_2(a))2\gamma_2 I_{CASE,1}(t,a,v_0) - 2\gamma_{3,0} I_{HOSPITAL,0}(t,a,v_0,1{,}0)$$

$$\frac{dI_{HOSPITAL,1}(t,a,v_0,1{,}0)}{dt} = 2\gamma_{3,0} I_{HOSPITAL,0}(t,a,v_0,1{,}0) - 2\gamma_{3,0} I_{HOSPITAL,1}(t,a,v_0,1{,}0)$$

$$\frac{dI_{HOSPITAL,0}(t,a,v_0,0{,}1)}{dt} = (1-\delta(H))(1-\mu(a))(1-\phi_2(a))2\gamma_2 I_{CASE,1}(t,a,v_0) - 2\gamma_{3,1} I_{HOSPITAL,0}(t,a,v_0,0{,}1)$$

$$\frac{dI_{HOSPITAL,1}(t,a,v_0,0{,}1)}{dt} = 2\gamma_{3,1} I_{HOSPITAL,0}(t,a,v_0,0{,}1) - 2\gamma_{3,1} I_{HOSPITAL,1}(t,a,v_0,0{,}1)$$

$$\frac{dI_{HOSPITAL,0}(t,a,v_0,1{,}1)}{dt} = \delta(H)(1-\mu(a))(1-\phi_2(a))2\gamma_2 I_{CASE,1}(t,a,v_0) - 2\gamma_{3,1} I_{HOSPITAL,0}(t,a,v_0,1{,}1)$$

$$\frac{dI_{HOSPITAL,1}(t,a,v_0,1{,}1)}{dt} = 2\gamma_{3,1} I_{HOSPITAL,0}(t,a,v_0,1{,}1) - 2\gamma_{3,1} I_{HOSPITAL,1}(t,a,v_0,1{,}1)$$

$$\frac{dI_{ICU,0}(t,a,v_0,0{,}0)}{dt} = (1-\delta(ICU))\mu(a)\phi_2(a)2\gamma_2 I_{CASE,1}(t,a,v_0) - 2\gamma_{4,0} I_{ICU,0}(t,a,v_0,0{,}0)$$

$$\frac{dI_{ICU,1}(t,a,v_0,0{,}0)}{dt} = 2\gamma_{4,0} I_{ICU,0}(t,a,v_0,0{,}0) - 2\gamma_{4,0} I_{ICU,1}(t,a,v_0,0{,}0)$$

$$\frac{dI_{ICU,0}(t,a,v_0,1{,}0)}{dt} = \delta(ICU)\mu(a)\phi_2(a)2\gamma_2 I_{CASE,1}(t,a,v_0) - 2\gamma_{4,0} I_{ICU,0}(t,a,v_0,1{,}0)$$

$$\frac{dI_{ICU,1}(t,a,v_0,1{,}0)}{dt} = 2\gamma_{4,0} I_{ICU,0}(t,a,v_0,1{,}0) - 2\gamma_{4,0} I_{ICU,1}(t,a,v_0,1{,}0)$$

$$\frac{dI_{ICU,0}(t,a,v_0,0{,}1)}{dt} = (1-\delta(ICU))(1-\mu(a))\phi_2(a)2\gamma_2 I_{CASE,1}(t,v_0,a) - 2\gamma_{4,1} I_{ICU,0}(t,a,v_0,0{,}1)$$

$$\frac{dI_{ICU,1}(t,a,v_0,0{,}1)}{dt} = 2\gamma_{4,1} I_{ICU,0}(t,a,v_0,0{,}1) - 2\gamma_{4,1} I_{ICU,1}(t,a,v_0,0{,}1)$$

$$\frac{dI_{ICU,0}(t,a,v_0,1{,}1)}{dt} = \delta(ICU)(1-\mu(a))\phi_2(a)2\gamma_2 I_{CASE,1}(t,a,v_0) - 2\gamma_{4,1} I_{ICU,0}(t,a,v_0,1{,}1)$$

$$\frac{dI_{ICU,1}(t,a,v_0,1{,}1)}{dt} = 2\gamma_{4,1} I_{ICU,0}(t,a,v_0,1{,}1) - 2\gamma_{4,1} I_{ICU,1}(t,a,v_0,1{,}1)$$

$$\frac{dI_{REC,0}(t,a,v_0)}{dt} = 2\gamma_{4,1} I_{ICU,1}(t,a,v_0,0{,}1) + 2\gamma_{4,1} I_{ICU,1}(t,a,v_0,1{,}1) - 2\gamma_5 I_{REC,0}(t,a,v_0)$$

$$\frac{dI_{REC,1}(t,a,v_0)}{dt} = 2\gamma_5 I_{REC,0}(t,a,v_0) - 2\gamma_5 I_{REC,1}(t,a,v_0)$$

$$\begin{aligned}\frac{dR_1(t,a,v_0)}{dt} = {} & \gamma_1 I_{MILD}(t,a,v_0) + 2\gamma_{3,1} I_{HOSPITAL,1}(t,a,v_0,0{,}1) + 2\gamma_{3,1} I_{HOSPITAL,1}(t,a,v_0,1{,}1) + 2\gamma_5 I_{REC,1}(t,a,v_0) \\ & + 2\gamma_{4,1} I_{ICU,1}(t,a,v_0,0{,}1) + 2\gamma_{4,1} I_{ICU,1}(t,a,v_0,1{,}1) - 2\rho R_1(t,a,v_0) - \kappa(a)R_1(t,a,v_0)\end{aligned}$$

$$\frac{dR_2(t,a,v_0)}{dt} = 2\rho R_1(t,a,v_0) - 2\rho R_2(t,a,v_0) - \kappa(a)R_2(t,a,v_0)$$

$$\begin{aligned}\frac{dD(t,a,v_0)}{dt} = {} & 2\gamma_{3,0} I_{HOSPITAL,1}(t,a,v_0,0{,}0) + 2\gamma_{3,0} I_{HOSPITAL,1}(t,a,v_0,1{,}0) + 2\gamma_{4,0} I_{ICU,1}(t,a,v_0,0{,}0) \\ & + 2\gamma_{4,0} I_{ICU,1}(t,a,v_0,1{,}0)\end{aligned}$$

1.4.2 Vaccination group v_1 - vaccinated but not yet protected (state 1)

$$\frac{dS(t,a,v_1)}{dt} = \kappa(a)S(t,a,v_0) + 2\rho R_2(t,a,v_1) - \beta\frac{S(t,a,v_1)}{N}\sum_{a'} c(a,a')[\sum_{v}(I_{MILD}(t,a',v) + I_{CASE}(t,a',v))] - 2\omega S(t,a,v_1)$$

$$\frac{dE_1(t,a,v_1)}{dt} = \kappa(a)E_1(t,a,v_0) + \beta\frac{S(t,a,v_1)}{N}\sum_{a'} c(a,a')[\sum_{v}(I_{MILD}(t,a',v) + I_{CASE}(t,a',v))] - 2\alpha E_1(t,a,v_1) - 2\omega E_1(t,a,v_1)$$

$$\frac{dE_2(t,a,v_1)}{dt} = \kappa(a)E_2(t,a,v_0) + 2\alpha E_1(t,a,v_1) - 2\alpha E_2(t,a,v_1) - 2\omega E_2(t,a,v_1)$$

$$\frac{dI_{MILD}(t,a,v_1)}{dt} = (1-\phi_1(a))(2\alpha E_2(t,a,v_1)) - \gamma_1 I_{MILD}(t,a,v_1) - 2\omega I_{MILD}(t,a,v_1)$$

$$\frac{dI_{CASE,0}(t,a,v_1)}{dt} = \phi_1(a)(2\alpha E_2(t,a,v_1)) - 2\gamma_2 I_{CASE,0}(t,a,v_1) - 2\omega I_{CASE,0}(t,a,v_1)$$

$$\frac{dI_{CASE,1}(t,a,v_1)}{dt} = 2\gamma_2 I_{CASE,0}(t,a,v_1) - 2\gamma_2 I_{CASE,1}(t,a,v_1) - 2\omega I_{CASE,1}(t,a,v_1)$$

$$\frac{dI_{HOSPITAL,0}(t,a,v_1,0,0)}{dt} = (1-\delta(H))\mu(a)(1-\phi_2(a))2\gamma_2 I_{CASE,1}(t,a,v_1) - 2\gamma_{3,0} I_{HOSPITAL,0}(t,a,v_1,0,0) - 2\omega I_{HOSPITAL,0}(t,a,v_1,0,0)$$

$$\frac{dI_{HOSPITAL,1}(t,a,v_1,0,0)}{dt} = 2\gamma_{3,0} I_{HOSPITAL,0}(t,a,v_1,0,0) - 2\gamma_{3,0} I_{HOSPITAL,1}(t,a,v_1,0,0) - 2\omega I_{HOSPITAL,1}(t,a,v_1,0,0)$$

$$\frac{dI_{HOSPITAL,0}(t,a,v_1,1,0)}{dt} = \delta(H)\mu(a)(1-\phi_2(a))2\gamma_2 I_{CASE,1}(t,a,v_1) - 2\gamma_{3,0} I_{HOSPITAL,0}(t,a,v_1,1,0) - 2\omega I_{HOSPITAL,0}(t,a,v_1,1,0)$$

$$\frac{dI_{HOSPITAL,1}(t,a,v_1,1,0)}{dt} = 2\gamma_{3,0} I_{HOSPITAL,0}(t,a,v_1,1,0) - 2\gamma_{3,0} I_{HOSPITAL,1}(t,a,v_1,1,0) - 2\omega I_{HOSPITAL,1}(t,a,v_1,1,0)$$

$$\frac{dI_{HOSPITAL,0}(t,a,v_1,0,1)}{dt} = (1-\delta(H))(1-\mu(a))(1-\phi_2(a))2\gamma_2 I_{CASE,1}(t,a,v_1) - 2\gamma_{3,1} I_{HOSPITAL,0}(t,a,v_1,0,1) - 2\omega I_{HOSPITAL,0}(t,a,v_1,0,1)$$

$$\frac{dI_{HOSPITAL,1}(t,a,v_1,0,1)}{dt} = 2\gamma_{3,1} I_{HOSPITAL,0}(t,a,v_1,0,1) - 2\gamma_{3,1} I_{HOSPITAL,1}(t,a,v_1,0,1) - 2\omega I_{HOSPITAL,1}(t,a,v_1,0,1)$$

$$\frac{dI_{HOSPITAL,0}(t,a,v_1,1,1)}{dt} = \delta(H)(1-\mu(a))(1-\phi_2(a))2\gamma_2 I_{CASE,1}(t,a,v_1) - 2\gamma_{3,1} I_{HOSPITAL,0}(t,a,v_1,1,1) - 2\omega I_{HOSPITAL,0}(t,a,v_1,1,1)$$

$$\frac{dI_{HOSPITAL,1}(t,a,v_1,1,1)}{dt} = 2\gamma_{3,1} I_{HOSPITAL,0}(t,a,v_1,1,1) - 2\gamma_{3,1} I_{HOSPITAL,1}(t,a,v_1,1,1) - 2\omega I_{HOSPITAL,1}(t,a,v_1,1,1)$$

$$\frac{dI_{ICU,0}(t,a,v_1,0,0)}{dt} = (1-\delta(ICU))\mu(a)\phi_2(a)2\gamma_2 I_{CASE,1}(t,a,v_1) - 2\gamma_{4,0} I_{ICU,0}(t,a,v_1,0,0) - 2\omega I_{ICU,0}(t,a,v_1,0,0)$$

$$\frac{dI_{ICU,1}(t,a,v_1,0,0)}{dt} = 2\gamma_{4,0} I_{ICU,0}(t,a,v_1,0,0) - 2\gamma_{4,0} I_{ICU,1}(t,a,v_1,0,0) - 2\omega I_{ICU,1}(t,a,v_1,0,0)$$

$$\frac{dI_{ICU,0}(t,a,v_1,1,0)}{dt} = \delta(ICU)\mu(a)\phi_2(a)2\gamma_2 I_{CASE,1}(t,a,v_1) - 2\gamma_{4,0} I_{ICU,0}(t,a,v_1,1,0) - 2\omega I_{ICU,0}(t,a,v_1,1,0)$$

$$\frac{dI_{ICU,1}(t,a,v_1,1,0)}{dt} = 2\gamma_{4,0} I_{ICU,0}(t,a,v_1,1,0) - 2\gamma_{4,0} I_{ICU,1}(t,a,v_1,1,0) - 2\omega I_{ICU,1}(t,a,v_1,1,0)$$

$$\frac{dI_{ICU,0}(t,a,v_1,0,1)}{dt} = (1-\delta(ICU))(1-\mu(a))\phi_2(a)2\gamma_2 I_{CASE,1}(t,v_1,a) - 2\gamma_{4,1} I_{ICU,0}(t,a,v_1,0,1) - 2\omega I_{ICU,0}(t,a,v_1,0,1)$$

$$\frac{dI_{ICU,1}(t,a,v_1,0,1)}{dt} = 2\gamma_{4,1} I_{ICU,0}(t,a,v_1,0,1) - 2\gamma_{4,1} I_{ICU,1}(t,a,v_1,0,1) - 2\omega I_{ICU,1}(t,a,v_1,0,1)$$

$$\frac{dI_{ICU,0}(t,a,v_1,1,1)}{dt} = \delta(ICU)(1-\mu(a))\phi_2(a)2\gamma_2 I_{CASE,1}(t,a,v_1) - 2\gamma_{4,1} I_{ICU,0}(t,a,v_1,1,1) - 2\omega I_{ICU,0}(t,a,v_1,1,1)$$

$$\frac{dI_{ICU,1}(t,a,v_1,1,1)}{dt} = 2\gamma_{4,1} I_{ICU,0}(t,a,v_1,1,1) - 2\gamma_{4,1} I_{ICU,1}(t,a,v_1,1,1) - 2\omega I_{ICU,1}(t,a,v_1,1,1)$$

$$\frac{dI_{REC,0}(t,a,v_1)}{dt} = 2\gamma_{4,1} I_{ICU,1}(t,a,v_1,0,1) + 2\gamma_{4,1} I_{ICU,1}(t,a,v_1,1,1) - 2\gamma_5 I_{REC,0}(t,a,v_1) - 2\omega I_{REC,0}(t,a,v_1)$$

$$\frac{dI_{REC,1}(t,a,v_1)}{dt} = 2\gamma_5 I_{REC,0}(t,a,v_1) - 2\gamma_5 I_{REC,1}(t,a,v_1) - 2\omega I_{REC,1}(t,a,v_1)$$

$$\frac{dR_1(t,a,v_1)}{dt} = \kappa(a)R_1(t,a,v_0) + \gamma_1 I_{MILD}(t,a,v_1) + 2\gamma_{3,1} I_{HOSPITAL,1}(t,a,v_1,0,1) + 2\gamma_{3,1} I_{HOSPITAL,1}(t,a,v_1,1,1) + 2\gamma_5 I_{REC,1}(t,a,v_1) + 2\gamma_{4,1} I_{ICU,1}(t,a,v_1,0,1) + 2\gamma_{4,1} I_{ICU,1}(t,a,v_1,1,1) - 2\rho R_1(t,a,v_1) - 2\omega R(t,a,v_1)$$

$$\frac{dR_2(t,a,v_1)}{dt} = \kappa(a)R_2(t,a,v_0) + 2\rho R_1(t,a,v_1) - 2\rho R_2(t,a,v_1)$$

$$\frac{dD(t,a,v_1)}{dt} = 2\gamma_{3,0} I_{HOSPITAL,1}(t,a,v_1,0,0) + 2\gamma_{3,0} I_{HOSPITAL,1}(t,a,v_1,1,0) + 2\gamma_{4,0} I_{ICU,1}(t,a,v_1,0,0) + 2\gamma_{4,0} I_{ICU,1}(t,a,v_1,1,0)$$

1.4.3 Vaccination group v₂ - vaccinated but not yet protected (state 2)

$$\frac{dS(t,a,v_2)}{dt} = 2\rho R_2(t,a,v_2) - \beta\frac{S(t,a,v_2)}{N}\sum_{a'} c(a,a')[\sum_{v}(I_{MILD}(t,a',v) + I_{CASE}(t,a',v))] + 2\omega S(t,a,v_1) - 2\omega S(t,a,v_2)$$

$$\frac{dE_1(t,a,v_2)}{dt} = \beta\frac{S(t,a,v_2)}{N}\sum_{a'} c(a,a')[\sum_{v}(I_{MILD}(t,a',v) + I_{CASE}(t,a',v))] - 2\alpha E_1(t,a,v_2) + 2\omega E_1(t,a,v_1) - 2\omega E_1(t,a,v_2)$$

$$\frac{dE_2(t,a,v_2)}{dt} = 2\alpha E_1(t,a,v_2) - 2\alpha E_2(t,a,v_2) + 2\omega E_2(t,a,v_1) - 2\omega E_2(t,a,v_2)$$

$$\frac{dI_{MILD}(t,a,v_2)}{dt} = (1-\phi_1(a))(2\alpha E_2(t,a,v_2)) - \gamma_1 I_{MILD}(t,a,v_2) + 2\omega I_{MILD}(t,a,v_1) - 2\omega I_{MILD}(t,a,v_2)$$

$$\frac{dI_{CASE,0}(t,a,v_2)}{dt} = \phi_1(a)(2\alpha E_2(t,a,v_2)) - 2\gamma_2 I_{CASE,0}(t,a,v_2) + 2\omega I_{CASE,0}(t,a,v_1) - 2\omega I_{CASE,0}(t,a,v_2)$$

$$\frac{dI_{CASE,1}(t,a,v_2)}{dt} = 2\gamma_2 I_{CASE,0}(t,a,v_2) - 2\gamma_2 I_{CASE,1}(t,a,v_2) + 2\omega I_{CASE,1}(t,a,v_1) - 2\omega I_{CASE,1}(t,a,v_2)$$

$$\frac{dI_{HOSPITAL,0}(t,a,v_2,0,0)}{dt} = (1-\delta(H))\mu(a)(1-\phi_2(a))2\gamma_2 I_{CASE,1}(t,a,v_2) - 2\gamma_{3,0} I_{HOSPITAL,0}(t,a,v_2,0,0) + 2\omega I_{HOSPITAL,0}(t,a,v_1,0,0) - 2\omega I_{HOSPITAL,0}(t,a,v_2,0,0)$$

$$\frac{dI_{HOSPITAL,1}(t,a,v_2,0,0)}{dt} = 2\gamma_{3,0} I_{HOSPITAL,0}(t,a,v_2,0,0) - 2\gamma_{3,0} I_{HOSPITAL,1}(t,a,v_2,0,0) + 2\omega I_{HOSPITAL,1}(t,a,v_1,0,0) - 2\omega I_{HOSPITAL,1}(t,a,v_2,0,0)$$

$$\frac{dI_{HOSPITAL,0}(t,a,v_2,1,0)}{dt} = \delta(H)\mu(a)(1-\phi_2(a))2\gamma_2 I_{CASE,1}(t,a,v_2) - 2\gamma_{3,0} I_{HOSPITAL,0}(t,a,v_2,1,0) + 2\omega I_{HOSPITAL,0}(t,a,v_1,1,0) - 2\omega I_{HOSPITAL,0}(t,a,v_2,1,0)$$

$$\frac{dI_{HOSPITAL,1}(t,a,v_2,1,0)}{dt} = 2\gamma_{3,0} I_{HOSPITAL,0}(t,a,v_2,1,0) - 2\gamma_{3,0} I_{HOSPITAL,1}(t,a,v_2,1,0) + 2\omega I_{HOSPITAL,1}(t,a,v_1,1,0) - 2\omega I_{HOSPITAL,1}(t,a,v_2,1,0)$$

$$\frac{dI_{HOSPITAL,0}(t,a,v_2,0,1)}{dt} = (1-\delta(H))(1-\mu(a))(1-\phi_2(a))2\gamma_2 I_{CASE,1}(t,a,v_2) - 2\gamma_{3,1} I_{HOSPITAL,0}(t,a,v_2,0,1) + 2\omega I_{HOSPITAL,0}(t,a,v_1,0,1) - 2\omega I_{HOSPITAL,0}(t,a,v_2,0,1)$$

$$\frac{dI_{HOSPITAL,1}(t,a,v_2,0,1)}{dt} = 2\gamma_{3,1} I_{HOSPITAL,0}(t,a,v_2,0,1) - 2\gamma_{3,1} I_{HOSPITAL,1}(t,a,v_2,0,1) + 2\omega I_{HOSPITAL,1}(t,a,v_1,0,1) - 2\omega I_{HOSPITAL,1}(t,a,v_2,0,1)$$

$$\frac{dI_{HOSPITAL,0}(t,a,v_2,1,1)}{dt} = \delta(H)(1-\mu(a))(1-\phi_2(a))2\gamma_2 I_{CASE,1}(t,a,v_2) - 2\gamma_{3,1} I_{HOSPITAL,0}(t,a,v_2,1,1) + 2\omega I_{HOSPITAL,0}(t,a,v_1,1,1) - 2\omega I_{HOSPITAL,0}(t,a,v_2,1,1)$$

$$\frac{dI_{HOSPITAL,1}(t,a,v_2,1,1)}{dt} = 2\gamma_{3,1} I_{HOSPITAL,0}(t,a,v_2,1,1) - 2\gamma_{3,1} I_{HOSPITAL,1}(t,a,v_2,1,1) + 2\omega I_{HOSPITAL,1}(t,a,v_1,1,1) - 2\omega I_{HOSPITAL,1}(t,a,v_2,1,1)$$

$$\frac{dI_{ICU,0}(t,a,v_2,0,0)}{dt} = (1-\delta(ICU))\mu(a)\phi_2(a)2\gamma_2 I_{CASE,1}(t,a,v_2) - 2\gamma_{4,0} I_{ICU,0}(t,a,v_2,0,0) + 2\omega I_{ICU,0}(t,a,v_1,0,0) - 2\omega I_{ICU,0}(t,a,v_2,0,0)$$

$$\frac{dI_{ICU,1}(t,a,v_2,0,0)}{dt} = 2\gamma_{4,0} I_{ICU,0}(t,a,v_2,0,0) - 2\gamma_{4,0} I_{ICU,1}(t,a,v_2,0,0) + 2\omega I_{ICU,1}(t,a,v_1,0,0) - 2\omega I_{ICU,1}(t,a,v_2,0,0)$$

$$\frac{dI_{ICU,0}(t,a,v_2,1,0)}{dt} = \delta(ICU)\mu(a)\phi_2(a)2\gamma_2 I_{CASE,1}(t,a,v_2) - 2\gamma_{4,0} I_{ICU,0}(t,a,v_2,1,0) + 2\omega I_{ICU,0}(t,a,v_1,1,0) - 2\omega I_{ICU,0}(t,a,v_2,1,0)$$

$$\frac{dI_{ICU,1}(t,a,v_2,1,0)}{dt} = 2\gamma_{4,0} I_{ICU,0}(t,a,v_2,1,0) - 2\gamma_{4,0} I_{ICU,1}(t,a,v_2,1,0) + 2\omega I_{ICU,1}(t,a,v_1,1,0) - 2\omega I_{ICU,1}(t,a,v_2,1,0)$$

$$\frac{dI_{ICU,0}(t,a,v_2,0,1)}{dt} = (1-\delta(ICU))(1-\mu(a))\phi_2(a)2\gamma_2 I_{CASE,1}(t,v_2,a) - 2\gamma_{4,1} I_{ICU,0}(t,a,v_2,0,1) + 2\omega I_{ICU,0}(t,a,v_1,0,1) - 2\omega I_{ICU,0}(t,a,v_2,0,1)$$

$$\frac{dI_{ICU,1}(t,a,v_2,0,1)}{dt} = 2\gamma_{4,1} I_{ICU,0}(t,a,v_2,0,1) - 2\gamma_{4,1} I_{ICU,1}(t,a,v_2,0,1) + 2\omega I_{ICU,1}(t,a,v_1,0,1) - 2\omega I_{ICU,1}(t,a,v_2,0,1)$$

$$\frac{dI_{ICU,0}(t,a,v_2,1,1)}{dt} = \delta(ICU)(1-\mu(a))\phi_2(a)2\gamma_2 I_{CASE,1}(t,a,v_2) - 2\gamma_{4,1} I_{ICU,0}(t,a,v_2,1,1) + 2\omega I_{ICU,0}(t,a,v_1,1,1) - 2\omega I_{ICU,0}(t,a,v_2,1,1)$$

$$\frac{dI_{ICU,1}(t,a,v_2,1,1)}{dt} = 2\gamma_{4,1} I_{ICU,0}(t,a,v_2,1,1) - 2\gamma_{4,1} I_{ICU,1}(t,a,v_2,1,1) + 2\omega I_{ICU,1}(t,a,v_1,1,1) - 2\omega I_{ICU,1}(t,a,v_2,1,1)$$

$$\frac{dI_{REC,0}(t,a,v_2)}{dt} = 2\gamma_{4,1} I_{ICU,1}(t,a,v_2,0,1) + 2\gamma_{4,1} I_{ICU,1}(t,a,v_2,1,1) - 2\gamma_5 I_{REC,0}(t,a,v_2) + 2\omega I_{REC,0}(t,a,v_1) - 2\omega I_{REC,0}(t,a,v_2)$$

$$\frac{dI_{REC,1}(t,a,v_2)}{dt} = 2\gamma_5 I_{REC,0}(t,a,v_2) - 2\gamma_5 I_{REC,1}(t,a,v_2) + 2\omega I_{REC,1}(t,a,v_1) - 2\omega I_{REC,1}(t,a,v_2)$$

$$\frac{dR_1(t,a,v_2)}{dt} = \gamma_1 I_{MILD}(t,a,v_2) + 2\gamma_{3,1} I_{HOSPITAL,1}(t,a,v_2,0,1) + 2\gamma_{3,1} I_{HOSPITAL,1}(t,a,v_2,1,1) + 2\gamma_5 I_{REC,1}(t,a,v_2) + 2\gamma_{4,1} I_{ICU,1}(t,a,v_2,0,1) + 2\gamma_{4,1} I_{ICU,1}(t,a,v_2,1,1) - 2\rho R_1(t,a,v_2) + 2\omega R_1(t,a,v_1) - 2\omega R_1(t,a,v_2)$$

$$\frac{dR_2(t,a,v_2)}{dt} = 2\rho R_1(t,a,v_2) - 2\rho R_2(t,a,v_2) + 2\omega R_2(t,a,v_1) - 2\omega R_2(t,a,v_2)$$

$$\frac{dD(t,a,v_2)}{dt} = 2\gamma_{3,0} I_{HOSPITAL,1}(t,a,v_2,0,0) + 2\gamma_{3,0} I_{HOSPITAL,1}(t,a,v_2,1,0) + 2\gamma_{4,0} I_{ICU,1}(t,a,v_2,0,0) + 2\gamma_{4,0} I_{ICU,1}(t,a,v_2,1,0)$$

1.4.4 Vaccination group v_3 - vaccinated and protected (state 1)

$$\frac{dS(t,a,v_3)}{dt} = 2\rho R_2(t,a,v_3) - \upsilon_{inf}(a)\beta\frac{S(t,a,v_3)}{N}\sum_{a'} c(a,a')[\sum_v \left(I_{MILD}(t,a',v) + I_{CASE}(t,a',v)\right)] + 2\omega S(t,a,v_2) - 2\psi S(t,a,v_3)$$

$$\frac{dE_1(t,a,v_3)}{dt} = \upsilon_{inf}(a)\beta\frac{S(t,a,v_3)}{N}\sum_{a'} c(a,a')[\sum_v (I_{MILD}(t,a',v) + I_{CASE}(t,a',v))] - 2\alpha E_1(t,a,v_2) + 2\omega E_1(t,a,v_2) - 2\psi E_1(t,a,v_3)$$

$$\frac{dE_2(t,a,v_3)}{dt} = 2\alpha E_1(t,a,v_3) - 2\alpha E_2(t,a,v_3) + 2\omega E_2(t,a,v_2) - 2\psi E_2(t,a,v_3)$$

$$\frac{dI_{MILD}(t,a,v_3)}{dt} = (1-\upsilon_{dis}(a)\phi_1(a))(2\alpha E_2(t,a,v_3)) - \gamma_1 I_{MILD}(t,a,v_3) + 2\omega I_{MILD}(t,a,v_2) - 2\psi I_{MILD}(t,a,v_3)$$

$$\frac{dI_{CASE,0}(t,a,v_3)}{dt} = \upsilon_{dis}(a)\phi_1(a)(2\alpha E_2(t,a,v_3)) - 2\gamma_2 I_{CASE,0}(t,a,v_3) + 2\omega I_{CASE,0}(t,a,v_2) - 2\psi I_{CASE,0}(t,a,v_3)$$

$$\frac{dI_{CASE,1}(t,a,v_3)}{dt} = 2\gamma_2 I_{CASE,0}(t,a,v_3) - 2\gamma_2 I_{CASE,1}(t,a,v_3) + 2\omega I_{CASE,1}(t,a,v_2) - 2\psi I_{CASE,1}(t,a,v_3)$$

$$\frac{dI_{HOSPITAL,0}(t,a,v_3,0,0)}{dt} = (1-\delta(H))\mu(a)(1-\phi_2(a))2\gamma_2 I_{CASE,1}(t,a,v_3) - 2\gamma_{3,0} I_{HOSPITAL,0}(t,a,v_3,0,0) + 2\omega I_{HOSPITAL,0}(t,a,v_2,0,0) - 2\psi I_{HOSPITAL,0}(t,a,v_3,0,0)$$

$$\frac{dI_{HOSPITAL,1}(t,a,v_3,0,0)}{dt} = 2\gamma_{3,0} I_{HOSPITAL,0}(t,a,v_3,0,0) - 2\gamma_{3,0} I_{HOSPITAL,1}(t,a,v_3,0,0) + 2\omega I_{HOSPITAL,1}(t,a,v_2,0,0) - 2\psi I_{HOSPITAL,1}(t,a,v_3,0,0)$$

$$\frac{dI_{HOSPITAL,0}(t,a,v_3,1,0)}{dt} = \delta(H)\mu(a)(1-\phi_2(a))2\gamma_2 I_{CASE,1}(t,a,v_3) - 2\gamma_{3,0} I_{HOSPITAL,0}(t,a,v_3,1,0) + 2\omega I_{HOSPITAL,0}(t,a,v_2,1,0) - 2\psi I_{HOSPITAL,0}(t,a,v_3,1,0)$$

$$\frac{dI_{HOSPITAL,1}(t,a,v_3,1,0)}{dt} = 2\gamma_{3,0} I_{HOSPITAL,0}(t,a,v_3,1,0) - 2\gamma_{3,0} I_{HOSPITAL,1}(t,a,v_3,1,0) + 2\omega I_{HOSPITAL,1}(t,a,v_2,1,0) - 2\psi I_{HOSPITAL,1}(t,a,v_3,1,0)$$

$$\frac{dI_{HOSPITAL,0}(t,a,v_3,0,1)}{dt} = (1-\delta(H))(1-\mu(a))(1-\phi_2(a))2\gamma_2 I_{CASE,1}(t,a,v_3) - 2\gamma_{3,1} I_{HOSPITAL,0}(t,a,v_3,0,1) + 2\omega I_{HOSPITAL,0}(t,a,v_2,0,1) - 2\psi I_{HOSPITAL,0}(t,a,v_3,0,1)$$

$$\frac{dI_{HOSPITAL,1}(t,a,v_3,0,1)}{dt} = 2\gamma_{3,1} I_{HOSPITAL,0}(t,a,v_3,0,1) - 2\gamma_{3,1} I_{HOSPITAL,1}(t,a,v_3,0,1) + 2\omega I_{HOSPITAL,1}(t,a,v_2,0,1) - 2\psi I_{HOSPITAL,1}(t,a,v_3,0,1)$$

$$\frac{dI_{HOSPITAL,0}(t,a,v_3,1,1)}{dt} = \delta(H)(1-\mu(a))(1-\phi_2(a))2\gamma_2 I_{CASE,1}(t,a,v_3) - 2\gamma_{3,1} I_{HOSPITAL,0}(t,a,v_3,1,1) + 2\omega I_{HOSPITAL,0}(t,a,v_2,1,1) - 2\psi I_{HOSPITAL,0}(t,a,v_3,1,1)$$

$$\frac{dI_{HOSPITAL,1}(t,a,v_3,1,1)}{dt} = 2\gamma_{3,1} I_{HOSPITAL,0}(t,a,v_3,1,1) - 2\gamma_{3,1} I_{HOSPITAL,1}(t,a,v_3,1,1) + 2\omega I_{HOSPITAL,1}(t,a,v_2,1,1) - 2\psi I_{HOSPITAL,1}(t,a,v_3,1,1)$$

$$\frac{dI_{ICU,0}(t,a,v_3,0,0)}{dt} = (1-\delta(ICU))\mu(a)\phi_2(a)2\gamma_2 I_{CASE,1}(t,a,v_3) - 2\gamma_{4,0} I_{ICU,0}(t,a,v_3,0,0) + 2\omega I_{ICU,0}(t,a,v_2,0,0) - 2\psi I_{ICU,0}(t,a,v_3,0,0)$$

$$\frac{dI_{ICU,1}(t,a,v_3,0,0)}{dt} = 2\gamma_{4,0} I_{ICU,0}(t,a,v_3,0,0) - 2\gamma_{4,0} I_{ICU,1}(t,a,v_3,0,0) + 2\omega I_{ICU,1}(t,a,v_2,0,0) - 2\psi I_{ICU,1}(t,a,v_3,0,0)$$

$$\frac{dI_{ICU,0}(t,a,v_3,1,0)}{dt} = \delta(ICU)\mu(a)\phi_2(a)2\gamma_2 I_{CASE,1}(t,a,v_3) - 2\gamma_{4,0}I_{ICU,0}(t,a,v_3,1,0) + 2\omega I_{ICU,0}(t,a,v_2,1,0) - 2\psi I_{ICU,0}(t,a,v_3,1,0)$$

$$\frac{dI_{ICU,1}(t,a,v_3,1,0)}{dt} = 2\gamma_{4,0}I_{ICU,0}(t,a,v_3,1,0) - 2\gamma_{4,0}I_{ICU,1}(t,a,v_3,1,0) + 2\omega I_{ICU,1}(t,a,v_2,1,0) - 2\psi I_{ICU,1}(t,a,v_3,1,0)$$

$$\frac{dI_{ICU,0}(t,a,v_3,0,1)}{dt} = (1-\delta(ICU))(1-\mu(a))\phi_2(a)2\gamma_2 I_{CASE,1}(t,v_3,a) - 2\gamma_{4,1}I_{ICU,0}(t,a,v_3,0,1) + 2\omega I_{ICU,0}(t,a,v_2,0,1) - 2\psi I_{ICU,0}(t,a,v_3,0,1)$$

$$\frac{dI_{ICU,1}(t,a,v_3,0,1)}{dt} = 2\gamma_{4,1}I_{ICU,0}(t,a,v_3,0,1) - 2\gamma_{4,1}I_{ICU,1}(t,a,v_3,0,1) + 2\omega I_{ICU,1}(t,a,v_2,0,1) - 2\psi I_{ICU,1}(t,a,v_3,0,1)$$

$$\frac{dI_{ICU,0}(t,a,v_3,1,1)}{dt} = \delta(ICU)(1-\mu(a))\phi_2(a)2\gamma_2 I_{CASE,1}(t,a,v_3) - 2\gamma_{4,1}I_{ICU,0}(t,a,v_3,1,1) + 2\omega I_{ICU,0}(t,a,v_2,1,1) - 2\psi I_{ICU,0}(t,a,v_3,1,1)$$

$$\frac{dI_{ICU,1}(t,a,v_3,1,1)}{dt} = 2\gamma_{4,1}I_{ICU,0}(t,a,v_3,1,1) - 2\gamma_{4,1}I_{ICU,1}(t,a,v_3,1,1) + 2\omega I_{ICU,1}(t,a,v_2,1,1) - 2\psi I_{ICU,1}(t,a,v_3,1,1)$$

$$\frac{dI_{REC,0}(t,a,v_3)}{dt} = 2\gamma_{4,1}I_{ICU,1}(t,a,v_3,0,1) + 2\gamma_{4,1}I_{ICU,1}(t,a,v_3,1,1) - 2\gamma_5 I_{REC,0}(t,a,v_3) + 2\omega I_{REC,0}(t,a,v_2) - 2\psi I_{REC,0}(t,a,v_3)$$

$$\frac{dI_{REC,1}(t,a,v_3)}{dt} = 2\gamma_5 I_{REC,0}(t,a,v_3) - 2\gamma_5 I_{REC,1}(t,a,v_3) + 2\omega I_{REC,1}(t,a,v_2) - 2\psi I_{REC,1}(t,a,v_3)$$

$$\frac{dR_1(t,a,v_3)}{dt} = \gamma_1 I_{MILD}(t,a,v_3) + 2\gamma_{3,1}I_{HOSPITAL,1}(t,a,v_3,0,1) + 2\gamma_{3,1}I_{HOSPITAL,1}(t,a,v_3,1,1) + 2\gamma_5 I_{REC,1}(t,a,v_3) + 2\gamma_{4,1}I_{ICU,1}(t,a,v_3,0,1) + 2\gamma_{4,1}I_{ICU,1}(t,a,v_3,1,1) - 2\rho R_1(t,a,v_3) + 2\omega R_1(t,a,v_2) - 2\psi R_1(t,a,v_3)$$

$$\frac{dR_2(t,a,v_3)}{dt} = 2\rho R_1(t,a,v_3) - 2\rho R_2(t,a,v_3) + 2\omega R_2(t,a,v_2) - 2\psi R_2(t,a,v_3)$$

$$\frac{dD(t,a,v_3)}{dt} = 2\gamma_{3,0}I_{HOSPITAL,1}(t,a,v_3,0,0) + 2\gamma_{3,0}I_{HOSPITAL,1}(t,a,v_3,1,0) + 2\gamma_{4,0}I_{ICU,1}(t,a,v_3,0,0) + 2\gamma_{4,0}I_{ICU,1}(t,a,v_3,1,0)$$

1.4.5 Vaccination group v_4 - vaccinated and protected (state 2)

$$\frac{dS(t,a,v_4)}{dt} = 2\rho R_2(t,a,v_4) - v_{inf}(a)\beta\frac{S(t,a,v_4)}{N}\sum_{a'} c(a,a')[\sum_{v}\left(I_{MILD}(t,a',v) + I_{CASE}(t,a',v)\right)] + 2\psi S(t,a,v_3) - 2\psi S(t,a,v_4)$$

$$\frac{dE_1(t,a,v_4)}{dt} = v_{inf}(a)\beta\frac{S(t,a,v_4)}{N}\sum_{a'} c(a,a')[\sum_{v}(I_{MILD}(t,a',v) + I_{CASE}(t,a',v))] - 2\alpha E_1(t,a,v_4) + 2\psi E_1(t,a,v_3) - 2\psi E_1(t,a,v_4)$$

$$\frac{dE_2(t,a,v_4)}{dt} = 2\alpha E_1(t,a,v_4) - 2\alpha E_2(t,a,v_4) + 2\psi E_2(t,a,v_3) - 2\psi E_2(t,a,v_4)$$

$$\frac{dI_{MILD}(t,a,v_4)}{dt} = (1 - v_{dis}(a)\phi_1(a))(2\alpha E_2(t,a,v_4)) - \gamma_1 I_{MILD}(t,a,v_4) + 2\psi I_{MILD}(t,a,v_3) - 2\psi I_{MILD}(t,a,v_4)$$

$$\frac{dI_{CASE,0}(t,a,v_4)}{dt} = v_{dis}(a)\phi_1(a)(2\alpha E_2(t,a,v_4)) - 2\gamma_2 I_{CASE,0}(t,a,v_4) + 2\psi I_{CASE,0}(t,a,v_3) - 2\psi I_{CASE,0}(t,a,v_4)$$

$$\frac{dI_{CASE,1}(t,a,v_4)}{dt} = 2\gamma_2 I_{CASE,0}(t,a,v_4) - 2\gamma_2 I_{CASE,1}(t,a,v_4) + 2\psi I_{CASE,1}(t,a,v_3) - 2\psi I_{CASE,1}(t,a,v_4)$$

$$\frac{dI_{HOSPITAL,0}(t,a,v_4,0,0)}{dt} = (1-\delta(H))\mu(a)(1-\phi_2(a))2\gamma_2 I_{CASE,1}(t,a,v_4) - 2\gamma_{3,0} I_{HOSPITAL,0}(t,a,v_4,0,0) + 2\psi I_{HOSPITAL,0}(t,a,v_3,0,0) - 2\psi I_{HOSPITAL,0}(t,a,v_4,0,0)$$

$$\frac{dI_{HOSPITAL,1}(t,a,v_4,0,0)}{dt} = 2\gamma_{3,0} I_{HOSPITAL,0}(t,a,v_4,0,0) - 2\gamma_{3,0} I_{HOSPITAL,1}(t,a,v_4,0,0) + 2\psi I_{HOSPITAL,1}(t,a,v_3,0,0) - 2\psi I_{HOSPITAL,1}(t,a,v_4,0,0)$$

$$\frac{dI_{HOSPITAL,0}(t,a,v_4,1,0)}{dt} = \delta(H)\mu(a)(1-\phi_2(a))2\gamma_2 I_{CASE,1}(t,a,v_4) - 2\gamma_{3,0} I_{HOSPITAL,0}(t,a,v_4,1,0) + 2\psi I_{HOSPITAL,0}(t,a,v_3,1,0) - 2\psi I_{HOSPITAL,0}(t,a,v_3,1,0)$$

$$\frac{dI_{HOSPITAL,1}(t,a,v_4,1,0)}{dt} = 2\gamma_{3,0} I_{HOSPITAL,0}(t,a,v_4,1,0) - 2\gamma_{3,0} I_{HOSPITAL,1}(t,a,v_4,1,0) + 2\psi I_{HOSPITAL,1}(t,a,v_3,1,0) - 2\psi I_{HOSPITAL,1}(t,a,v_4,1,0)$$

$$\frac{dI_{HOSPITAL,0}(t,a,v_4,0,1)}{dt} = (1-\delta(H))(1-\mu(a))(1-\phi_2(a))2\gamma_2 I_{CASE,1}(t,a,v_4) - 2\gamma_{3,1} I_{HOSPITAL,0}(t,a,v_4,0,1) + 2\psi I_{HOSPITAL,0}(t,a,v_3,0,1) - 2\psi I_{HOSPITAL,0}(t,a,v_4,0,1)$$

$$\frac{dI_{HOSPITAL,1}(t,a,v_4,0,1)}{dt} = 2\gamma_{3,1} I_{HOSPITAL,0}(t,a,v_4,0,1) - 2\gamma_{3,1} I_{HOSPITAL,1}(t,a,v_4,0,1) + 2\psi I_{HOSPITAL,1}(t,a,v_3,0,1) - 2\psi I_{HOSPITAL,1}(t,a,v_4,0,1)$$

$$\frac{dI_{HOSPITAL,0}(t,a,v_4,1,1)}{dt} = \delta(H)(1-\mu(a))(1-\phi_2(a))2\gamma_2 I_{CASE,1}(t,a,v_4) - 2\gamma_{3,1} I_{HOSPITAL,0}(t,a,v_4,1,1) + 2\psi I_{HOSPITAL,0}(t,a,v_3,1,1) - 2\psi I_{HOSPITAL,0}(t,a,v_4,1,1)$$

$$\frac{dI_{HOSPITAL,1}(t,a,v_4,1,1)}{dt} = 2\gamma_{3,1} I_{HOSPITAL,0}(t,a,v_4,1,1) - 2\gamma_{3,1} I_{HOSPITAL,1}(t,a,v_4,1,1) + 2\psi I_{HOSPITAL,1}(t,a,v_3,1,1) - 2\psi I_{HOSPITAL,1}(t,a,v_4,1,1)$$

$$\frac{dI_{ICU,0}(t,a,v_4,0,0)}{dt} = (1-\delta(ICU))\mu(a)\phi_2(a)2\gamma_2 I_{CASE,1}(t,a,v_4) - 2\gamma_{4,0} I_{ICU,0}(t,a,v_4,0,0) + 2\psi I_{ICU,0}(t,a,v_3,0,0) - 2\psi I_{ICU,0}(t,a,v_4,0,0)$$

$$\frac{dI_{ICU,1}(t,a,v_4,0,0)}{dt} = 2\gamma_{4,0} I_{ICU,0}(t,a,v_4,0,0) - 2\gamma_{4,0} I_{ICU,1}(t,a,v_4,0,0) + 2\psi I_{ICU,1}(t,a,v_3,0,0) - 2\psi I_{ICU,1}(t,a,v_4,0,0)$$

$$\frac{dI_{ICU,0}(t,a,v_4,1,0)}{dt} = \delta(ICU)\mu(a)\phi_2(a)2\gamma_2 I_{CASE,1}(t,a,v_4) - 2\gamma_{4,0} I_{ICU,0}(t,a,v_4,1,0) + 2\psi I_{ICU,0}(t,a,v_3,1,0) - 2\psi I_{ICU,0}(t,a,v_4,1,0)$$

$$\frac{dI_{ICU,1}(t,a,v_4,1,0)}{dt} = 2\gamma_{4,0} I_{ICU,0}(t,a,v_4,1,0) - 2\gamma_{4,0} I_{ICU,1}(t,a,v_4,1,0) + 2\psi I_{ICU,1}(t,a,v_3,1,0) - 2\psi I_{ICU,1}(t,a,v_4,1,0)$$

$$\frac{dI_{ICU,0}(t,a,v_4,0,1)}{dt} = (1-\delta(ICU))(1-\mu(a))\phi_2(a)2\gamma_2 I_{CASE,1}(t,v_4,a) - 2\gamma_{4,1} I_{ICU,0}(t,a,v_4,0,1) + 2\psi I_{ICU,0}(t,a,v_3,0,1) - 2\psi I_{ICU,0}(t,a,v_4,0,1)$$

$$\frac{dI_{ICU,1}(t,a,v_4,0,1)}{dt} = 2\gamma_{4,1} I_{ICU,0}(t,a,v_4,0,1) - 2\gamma_{4,1} I_{ICU,1}(t,a,v_4,0,1) + 2\psi I_{ICU,1}(t,a,v_3,0,1) - 2\psi I_{ICU,1}(t,a,v_4,0,1)$$

$$\frac{dI_{ICU,0}(t,a,v_4,1,1)}{dt} = \delta(ICU)(1-\mu(a))\phi_2(a)2\gamma_2 I_{CASE,1}(t,a,v_4) - 2\gamma_{4,1} I_{ICU,0}(t,a,v_4,1,1) + 2\psi I_{ICU,0}(t,a,v_3,1,1) - 2\psi I_{ICU,0}(t,a,v_4,1,1)$$

$$\frac{dI_{ICU,1}(t,a,v_4,1,1)}{dt} = 2\gamma_{4,1} I_{ICU,0}(t,a,v_4,1,1) - 2\gamma_{4,1} I_{ICU,1}(t,a,v_4,1,1) + 2\psi I_{ICU,1}(t,a,v_3,1,1) - 2\psi I_{ICU,1}(t,a,v_4,1,1)$$

$$\frac{dI_{REC,0}(t,a,v_4)}{dt} = 2\gamma_{4,1} I_{ICU,1}(t,a,v_4,0,1) + 2\gamma_{4,1} I_{ICU,1}(t,a,v_4,1,1) - 2\gamma_5 I_{REC,0}(t,a,v_4) + 2\psi I_{REC,0}(t,a,v_3) - 2\psi I_{REC,0}(t,a,v_4)$$

$$\frac{dI_{REC,1}(t,a,v_4)}{dt} = 2\gamma_5 I_{REC,0}(t,a,v_4) - 2\gamma_5 I_{REC,1}(t,a,v_4) + 2\psi I_{REC,1}(t,a,v_3) - 2\psi I_{REC,1}(t,a,v_4)$$

$$\frac{dR_1(t,a,v_4)}{dt} = \gamma_1 I_{MILD}(t,a,v_4) + 2\gamma_{3,1} I_{HOSPITAL,1}(t,a,v_4,0,1) + 2\gamma_{3,1} I_{HOSPITAL,1}(t,a,v_4,1,1) + 2\gamma_5 I_{REC,1}(t,a,v_4) + 2\gamma_{4,1} I_{ICU,1}(t,a,v_4,0,1) + 2\gamma_{4,1} I_{ICU,1}(t,a,v_4,1,1) - 2\rho R_1(t,a,v_4) + 2\psi R_1(t,a,v_3) - 2\psi R_1(t,a,v_4)$$

$$\frac{dR_2(t,a,v_4)}{dt} = 2\rho R_1(t,a,v_4) - 2\rho R_2(t,a,v_4) + 2\psi R_2(t,a,v_3) - 2\psi R_2(t,a,v_4)$$

$$\frac{dD(t,a,v_4)}{dt} = 2\gamma_{3,0} I_{HOSPITAL,1}(t,a,v_4,0,0) + 2\gamma_{3,0} I_{HOSPITAL,1}(t,a,v_4,1,0) + 2\gamma_{4,0} I_{ICU,1}(t,a,v_4,0,0) + 2\gamma_{4,0} I_{ICU,1}(t,a,v_4,1,0)$$

1.4.6 Vaccination group v_5 - previously vaccinated but no longer protected

$$\frac{dS(t,a,v_5)}{dt} = 2\rho R_2(t,a,v_5) - \beta\frac{S(t,a,v_5)}{N}\sum_{a'} c(a,a')[\sum_{v}(I_{MILD}(t,a',v) + I_{CASE}(t,a',v))] + 2\psi S(t,a,v_4)$$

$$\frac{dE_1(t,a,v_5)}{dt} = \beta\frac{S(t,a,v_5)}{N}\sum_{a'} c(a,a')[\sum_{v}(I_{MILD}(t,a',v) + I_{CASE}(t,a',v))] - 2\alpha E_1(t,a,v_5) + 2\psi E_1(t,a,v_4)$$

$$\frac{dE_2(t,a,v_5)}{dt} = 2\alpha E_1(t,a,v_5) - 2\alpha E_2(t,a,v_5) + 2\psi E_2(t,a,v_4)$$

$$\frac{dI_{MILD}(t,a,v_5)}{dt} = (1-\phi_1(a))(2\alpha E_2(t,a,v_5)) - \gamma_1 I_{MILD}(t,a,v_5) + 2\psi I_{MILD}(t,a,v_4)$$

$$\frac{dI_{CASE,0}(t,a,v_5)}{dt} = \phi_1(a)(2\alpha E_2(t,a,v_5)) - 2\gamma_2 I_{CASE,0}(t,a,v_5) + 2\psi I_{CASE,0}(t,a,v_4)$$

$$\frac{dI_{CASE,1}(t,a,v_5)}{dt} = 2\gamma_2 I_{CASE,0}(t,a,v_5) - 2\gamma_2 I_{CASE,1}(t,a,v_5) + 2\psi I_{CASE,1}(t,a,v_4)$$

$$\frac{dI_{HOSPITAL,0}(t,a,v_5,0{,}0)}{dt} = (1-\delta(H))\mu(a)(1-\phi_2(a))2\gamma_2 I_{CASE,1}(t,a,v_5) - 2\gamma_{3,0} I_{HOSPITAL,0}(t,a,v_5,0{,}0) + 2\psi I_{HOSPITAL,0}(t,a,v_4,0{,}0)$$

$$\frac{dI_{HOSPITAL,1}(t,a,v_5,0{,}0)}{dt} = 2\gamma_{3,0} I_{HOSPITAL,0}(t,a,v_5,0{,}0) - 2\gamma_{3,0} I_{HOSPITAL,1}(t,a,v_5,0{,}0) + 2\psi I_{HOSPITAL,1}(t,a,v_4,0{,}0)$$

$$\frac{dI_{HOSPITAL,0}(t,a,v_5,1{,}0)}{dt} = \delta(H)\mu(a)(1-\phi_2(a))2\gamma_2 I_{CASE,1}(t,a,v_5) - 2\gamma_{3,0} I_{HOSPITAL,0}(t,a,v_5,1{,}0) + 2\psi I_{HOSPITAL,0}(t,a,v_3,1{,}0)$$

$$\frac{dI_{HOSPITAL,1}(t,a,v_5,1{,}0)}{dt} = 2\gamma_{3,0} I_{HOSPITAL,0}(t,a,v_5,1{,}0) - 2\gamma_{3,0} I_{HOSPITAL,1}(t,a,v_5,1{,}0) + 2\psi I_{HOSPITAL,1}(t,a,v_4,1{,}0)$$

$$\frac{dI_{HOSPITAL,0}(t,a,v_5,0{,}1)}{dt} = (1-\delta(H))(1-\mu(a))(1-\phi_2(a))2\gamma_2 I_{CASE,1}(t,a,v_5) - 2\gamma_{3,1} I_{HOSPITAL,0}(t,a,v_5,0{,}1) + 2\psi I_{HOSPITAL,0}(t,a,v_4,0{,}1)$$

$$\frac{dI_{HOSPITAL,1}(t,a,v_5,0{,}1)}{dt} = 2\gamma_{3,1} I_{HOSPITAL,0}(t,a,v_5,0{,}1) - 2\gamma_{3,1} I_{HOSPITAL,1}(t,a,v_5,0{,}1) + 2\psi I_{HOSPITAL,1}(t,a,v_4,0{,}1)$$

$$\frac{dI_{HOSPITAL,0}(t,a,v_5,1{,}1)}{dt} = \delta(H)(1-\mu(a))(1-\phi_2(a))2\gamma_2 I_{CASE,1}(t,a,v_5) - 2\gamma_{3,1} I_{HOSPITAL,0}(t,a,v_5,1{,}1) + 2\psi I_{HOSPITAL,0}(t,a,v_4,1{,}1)$$

$$\frac{dI_{HOSPITAL,1}(t,a,v_5,1{,}1)}{dt} = 2\gamma_{3,1} I_{HOSPITAL,0}(t,a,v_5,1{,}1) - 2\gamma_{3,1} I_{HOSPITAL,1}(t,a,v_5,1{,}1) + 2\psi I_{HOSPITAL,1}(t,a,v_4,1{,}1)$$

$$\frac{dI_{ICU,0}(t,a,v_5,0{,}0)}{dt} = (1-\delta(ICU))\mu(a)\phi_2(a)2\gamma_2 I_{CASE,1}(t,a,v_5) - 2\gamma_{4,0} I_{ICU,0}(t,a,v_5,0{,}0) + 2\psi I_{ICU,0}(t,a,v_4,0{,}0)$$

$$\frac{dI_{ICU,1}(t,a,v_5,0{,}0)}{dt} = 2\gamma_{4,0} I_{ICU,0}(t,a,v_5,0{,}0) - 2\gamma_{4,0} I_{ICU,1}(t,a,v_5,0{,}0) + 2\psi I_{ICU,1}(t,a,v_4,0{,}0)$$

$$\frac{dI_{ICU,0}(t,a,v_5,1{,}0)}{dt} = \delta(ICU)\mu(a)\phi_2(a)2\gamma_2 I_{CASE,1}(t,a,v_5) - 2\gamma_{4,0} I_{ICU,0}(t,a,v_5,1{,}0) + 2\psi I_{ICU,0}(t,a,v_4,1{,}0)$$

$$\frac{dI_{ICU,1}(t,a,v_5,1{,}0)}{dt} = 2\gamma_{4,0} I_{ICU,0}(t,a,v_5,1{,}0) - 2\gamma_{4,0} I_{ICU,1}(t,a,v_5,1{,}0) + 2\psi I_{ICU,1}(t,a,v_4,1{,}0)$$

$$\frac{dI_{ICU,0}(t,a,v_5,0{,}1)}{dt} = (1-\delta(ICU))(1-\mu(a))\phi_2(a)2\gamma_2 I_{CASE,1}(t,v_5,a) - 2\gamma_{4,1} I_{ICU,0}(t,a,v_5,0{,}1) + 2\psi I_{ICU,0}(t,a,v_4,0{,}1)$$

$$\frac{dI_{ICU,1}(t,a,v_5,0{,}1)}{dt} = 2\gamma_{4,1} I_{ICU,0}(t,a,v_5,0{,}1) - 2\gamma_{4,1} I_{ICU,1}(t,a,v_5,0{,}1) + 2\psi I_{ICU,1}(t,a,v_4,0{,}1)$$

$$\frac{dI_{ICU,0}(t,a,v_5,1{,}1)}{dt} = \delta(ICU)(1-\mu(a))\phi_2(a)2\gamma_2 I_{CASE,1}(t,a,v_5) - 2\gamma_{4,1} I_{ICU,0}(t,a,v_5,1{,}1) + 2\psi I_{ICU,0}(t,a,v_4,1{,}1)$$

$$\frac{dI_{ICU,1}(t,a,v_5,1{,}1)}{dt} = 2\gamma_{4,1} I_{ICU,0}(t,a,v_5,1{,}1) - 2\gamma_{4,1} I_{ICU,1}(t,a,v_5,1{,}1) + 2\psi I_{ICU,1}(t,a,v_4,1{,}1)$$

$$\frac{dI_{REC,0}(t,a,v_5)}{dt} = 2\gamma_{4,1} I_{ICU,1}(t,a,v_5,0{,}1) + 2\gamma_{4,1} I_{ICU,1}(t,a,v_5,1{,}1) - 2\gamma_5 I_{REC,0}(t,a,v_5) + 2\psi I_{REC,0}(t,a,v_4)$$

$$\frac{dI_{REC,1}(t,a,v_5)}{dt} = 2\gamma_5 I_{REC,0}(t,a,v_5) - 2\gamma_5 I_{REC,1}(t,a,v_5) + 2\psi I_{REC,1}(t,a,v_4)$$

$$\frac{dR_1(t,a,v_5)}{dt} = \gamma_1 I_{MILD}(t,a,v_5) + 2\gamma_{3,1} I_{HOSPITAL,1}(t,a,v_5,0,1) + 2\gamma_{3,1} I_{HOSPITAL,1}(t,a,v_5,1,1) + 2\gamma_5 I_{REC,1}(t,a,v_5) + 2\gamma_{4,1} I_{ICU,1}(t,a,v_5,0,1) + 2\gamma_{4,1} I_{ICU,1}(t,a,v_5,1,1) - 2\rho R_1(t,a,v_5) + 2\psi R_1(t,a,v_4)$$

$$\frac{dR_2(t,a,v_5)}{dt} = 2\rho R_1(t,a,v_5) - 2\rho R_2(t,a,v_5) + 2\psi R_2(t,a,v_4)$$

$$\frac{dD(t,a,v_5)}{dt} = 2\gamma_{3,0} I_{HOSPITAL,1}(t,a,v_5,0,0) + 2\gamma_{3,0} I_{HOSPITAL,1}(t,a,v_5,1,0) + 2\gamma_{4,0} I_{ICU,1}(t,a,v_5,0,0) + 2\gamma_{4,0} I_{ICU,1}(t,a,v_5,1,0)$$

2 Additional Figures

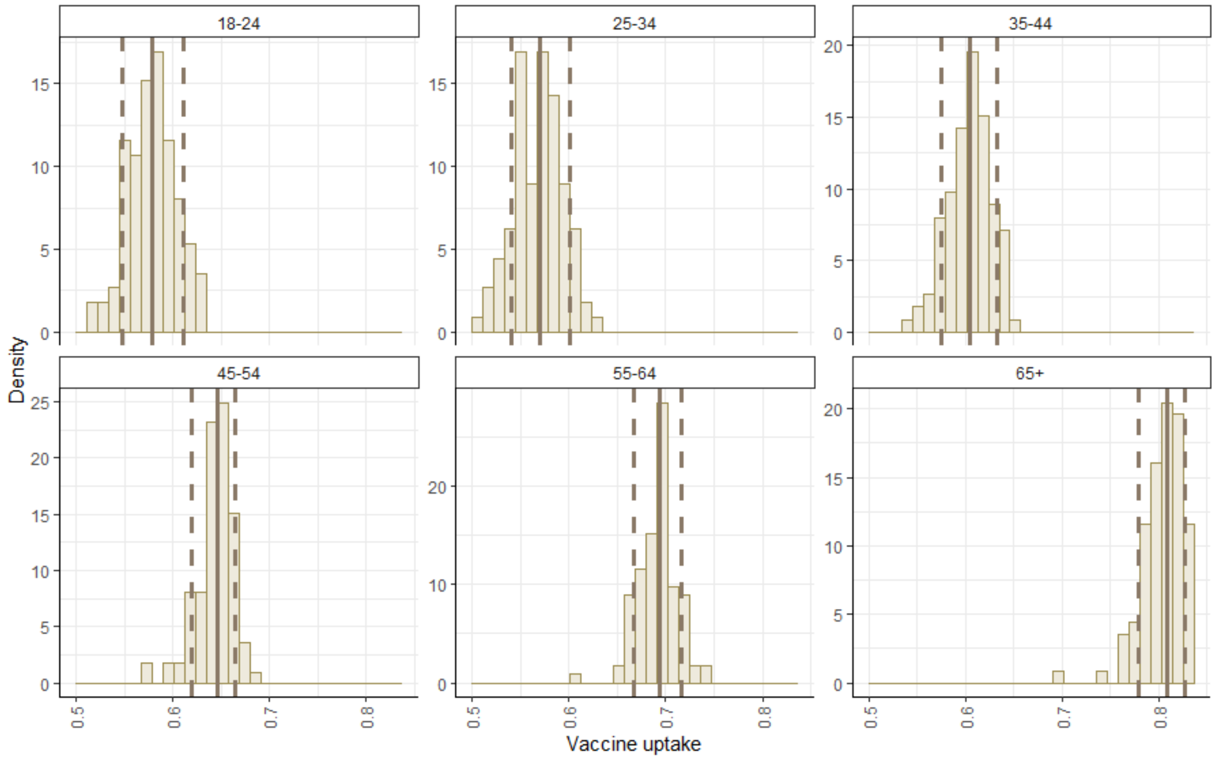

Figure S2 Vaccine uptake distribution per age group. Based on vaccine acceptance reported by Jones et al [31] distribution of coverage per age group was estimated after translating survey results into multinomial distribution and drawing 100 replicates. Probability of vaccination was modelled as a beta distribution with mean 0.98, 0.75,0.50,0.25 and 0.02 for survey response 1,2,3,4,5, respectively.

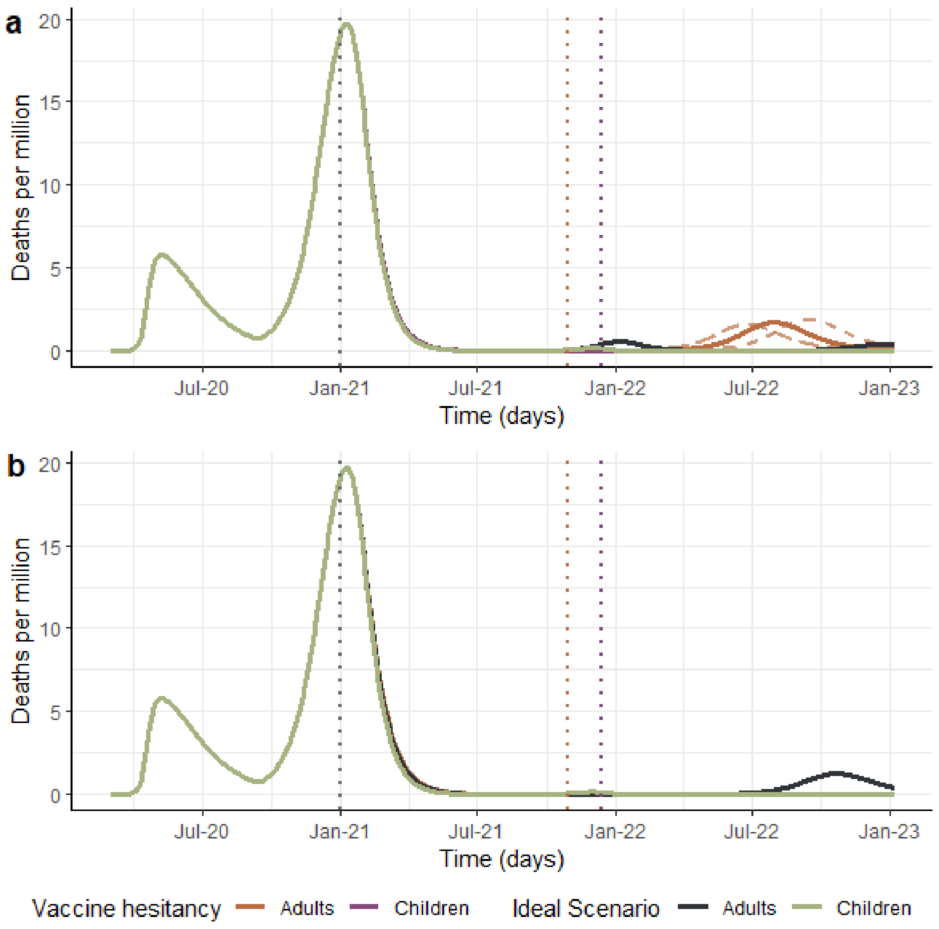

Figure S3 - COVID-19 dynamics for different reproductive number profiles. Profiles were estimated for each vaccine hesitancy scenario in order to achieve herd immunity and control the pandemic. **a)** Daily projected deaths per million for a high vaccine efficacy. **b)** Daily projected deaths per million for a moderate vaccine efficacy. Orange shows a scenario with vaccine hesitancy and adults-only vaccination. Purple shows scenario with vaccine hesitancy and vaccination including children Ideal scenario. Ideal scenario for adults-only vaccination is shown with a black line shows and for a vaccination including children is shown with a green line. Continuous lines show results for median vaccine coverage per age group and dashed lines show results for 10% and 90% quantiles.

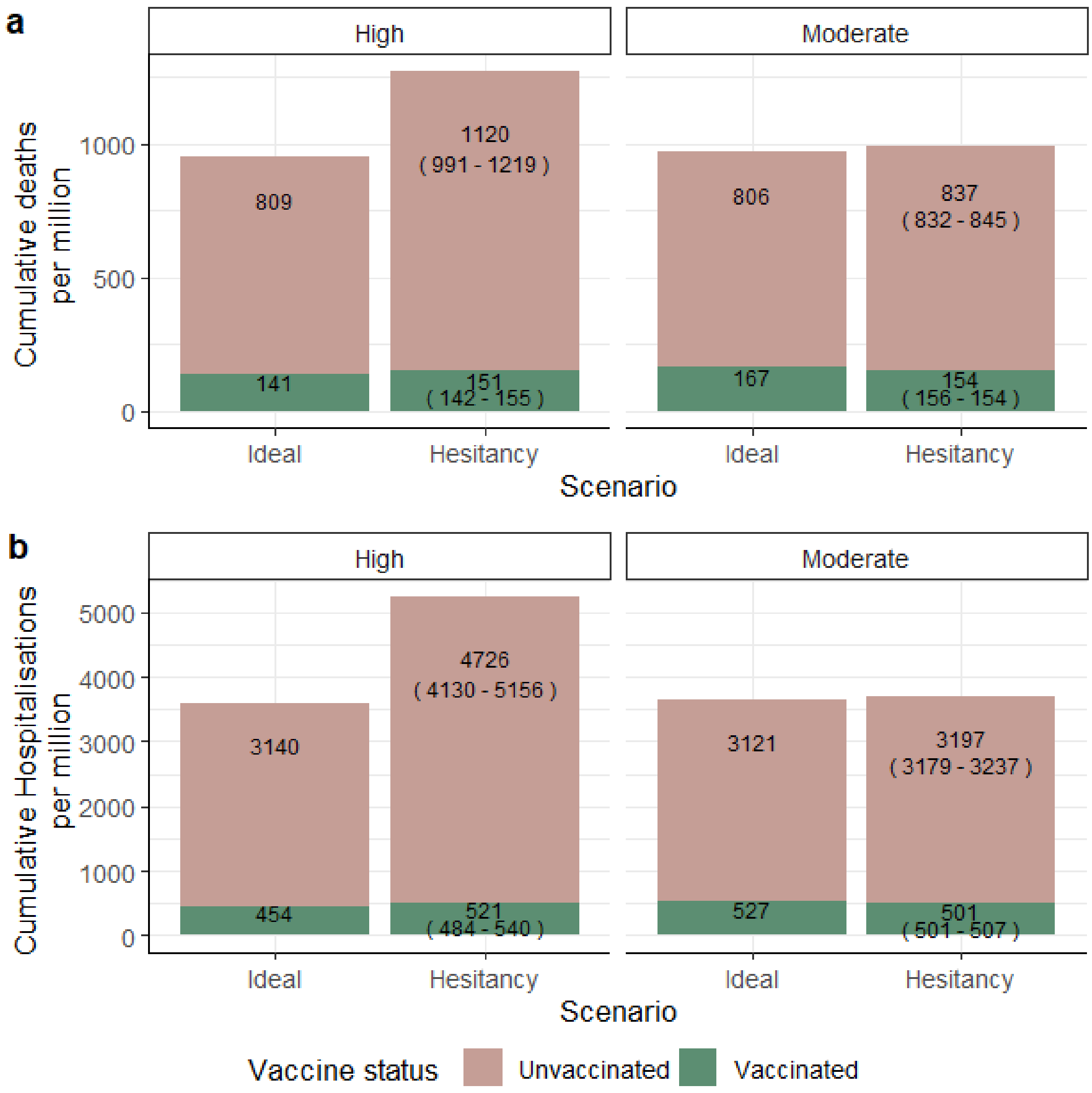

Figure S4. Public health impact of vaccine hesitancy for a vaccine roll out including children. High vaccine efficacy is shown on the left and moderate vaccine efficacy on the right**.** The annotated numbers are the cumulative deaths **(a)** and hospitalisations **(b)** per million individuals for the vaccinated and unvaccinated populations at the end of the projection horizon (1 January 2021 - 31 December 2022). Vaccination coverage of individuals aged 5 years and older is highest in the ideal scenario at 95%. For the hesitancy scenario annotated number is for median vaccine coverage per age groups, number in parenthesis are results for 10% and 90% quantiles coverage per age group.

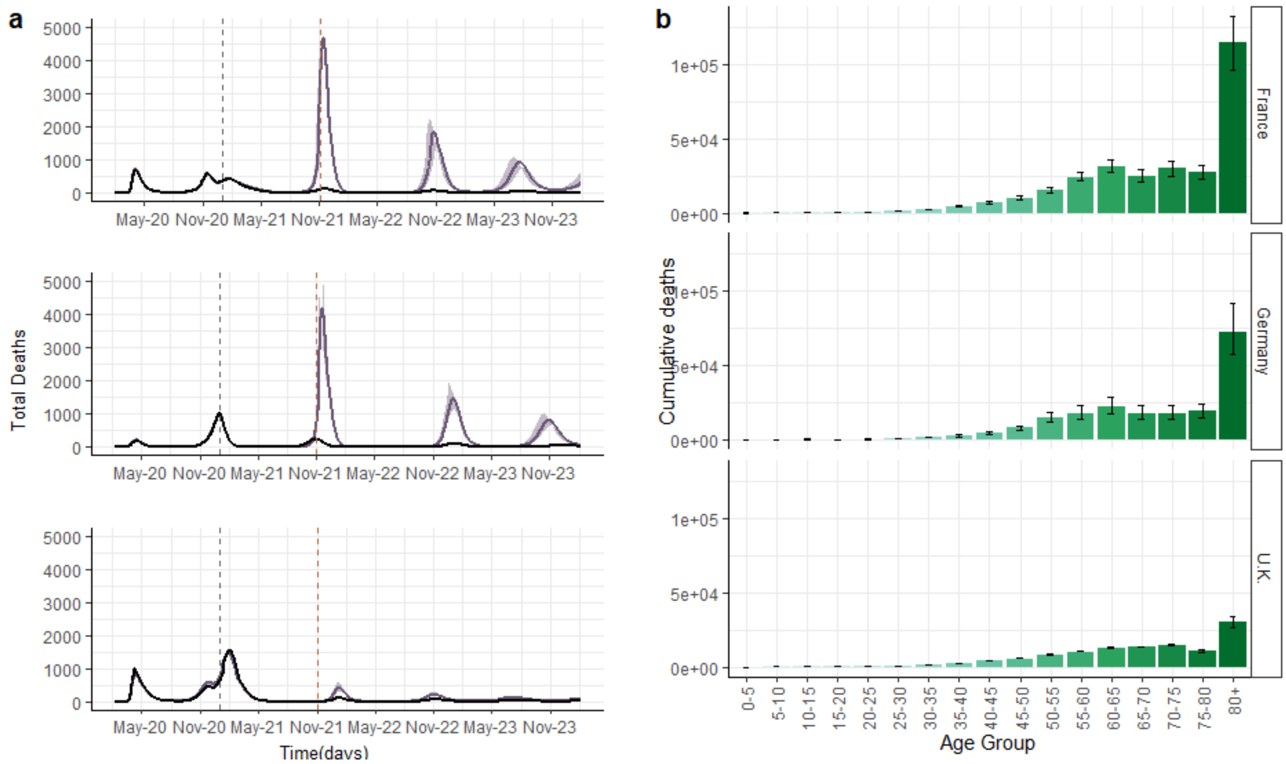

Figure S5. Predicted COVID-19 dynamics for each country for a high efficacy vaccine. a) Daily projected deaths per million. Black line shows an ideal scenario without vaccine hesitancy and 95% of individuals above 15 years old, are vaccinated. Purple shows scenario with vaccine hesitancy. Continuous lines show results for median vaccine coverage per age group and shadowed area show upper and lower bounds for 10% and 90% quantile for vaccination coverage. **b)** Total deaths per age group for median vaccination coverage. Total deaths are estimated over a two-year period since vaccination starts. Vertical dotted lines show the period of vaccination in the ideal scenario.

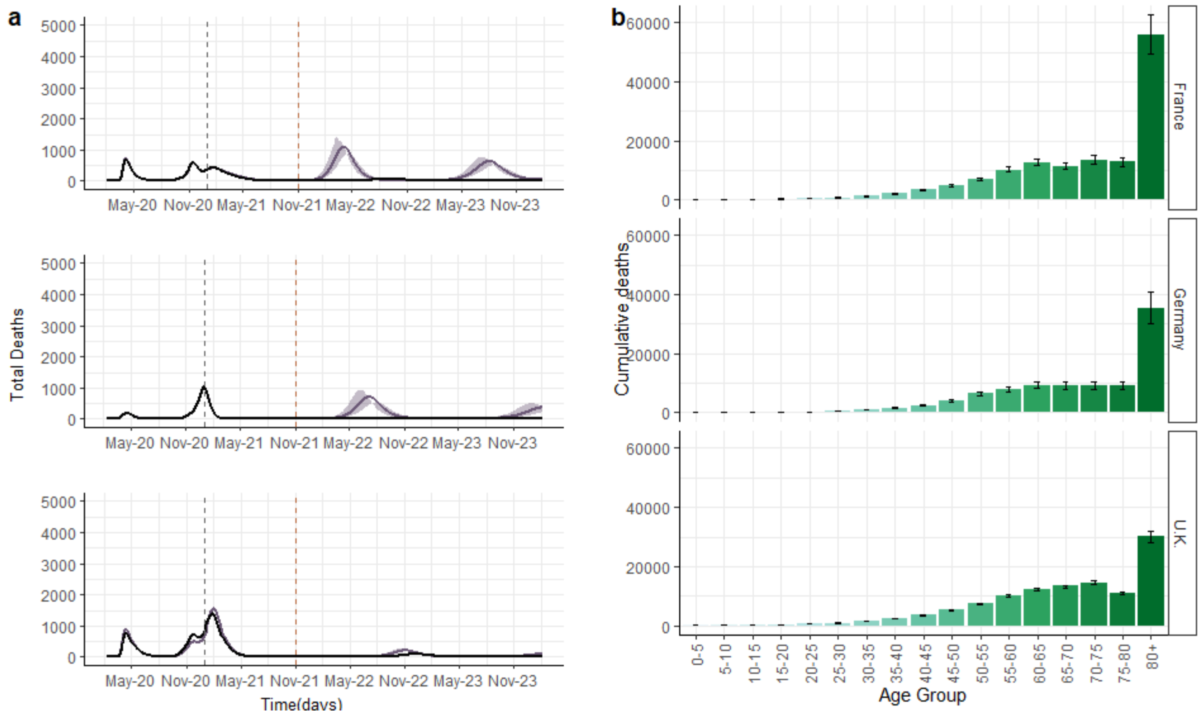

Figure S6. Predicted COVID-19 dynamics for each country for a moderate efficacy vaccine. a) Daily projected deaths per million. Black line shows an ideal scenario without vaccine hesitancy and 95% of individuals above 15 years old, are vaccinated. Purple shows scenario with vaccine hesitancy. Continuous lines show results for median vaccine coverage per age group and shadowed area show upper and lower bounds for 10% and 90% quantile vaccination coverage. **b)** Total deaths per age group for median vaccination coverage. Total deaths are estimated over a two-year period since vaccination starts. Vertical dotted lines show the period of vaccination in the ideal scenario.

References

1. Hogan, A.B., *et al.* Within-country age-based prioritisation, global allocation, and public health impact of a vaccine against SARS-CoV-2: A mathematical modelling analysis. *Vaccine* (2021).
2. Walker, P.G.T., *et al.* The impact of COVID-19 and strategies for mitigation and suppression in low- and middle-income countries. *Science* **369**, 413-422 (2020).
3. Linton, N.M., *et al.* Incubation Period and Other Epidemiological Characteristics of 2019 Novel Coronavirus Infections with Right Truncation: A Statistical Analysis of Publicly Available Case Data. *J Clin Med* **9**, 538 (2020).
4. Li, Q., *et al.* Early Transmission Dynamics in Wuhan, China, of Novel Coronavirus–Infected Pneumonia. *New England Journal of Medicine* **382**, 1199-1207 (2020).
5. Lauer, S.A., *et al.* The Incubation Period of Coronavirus Disease 2019 (COVID-19) From Publicly Reported Confirmed Cases: Estimation and Application. *Ann Intern Med* **172**, 577-582 (2020).
6. Bi, Q., *et al.* Epidemiology and transmission of COVID-19 in 391 cases and 1286 of their close contacts in Shenzhen, China: a retrospective cohort study. *The Lancet Infectious Diseases* **20**, 911-919 (2020).
7. Docherty, A.B., *et al.* Features of 20 133 UK patients in hospital with covid-19 using the ISARIC WHO Clinical Characterisation Protocol: prospective observational cohort study. *BMJ* **369**, m1985 (2020).
8. Hawryluk, I., *et al.* Inference of COVID-19 epidemiological distributions from Brazilian hospital data. *J. R. Soc. Interface* **17**(2020).
9. South African COVID-19 Modelling Consortium X. Estimating cases for COVID-19 in South Africa. Short term Projections June 2020. (2020).
10. Sreevalsan-Nair, J., Vangimalla, R.R. & Ghogale, P.R. Analysis and Estimation of Length of In-Hospital Stay Using Demographic Data of COVID-19 Recovered Patients in Singapore. *medRxiv*, 2020.2004.2017.20069724 (2020).
11. Haw, N.J.L., Uy, J., Sy, K.T.L. & Abrigo, M.R.M. Epidemiological profile and transmission dynamics of COVID-19 in the Philippines. *Epidemiol Infect* **148**, e204-e204 (2020).
12. Oliveira, E., *et al.* ICU Outcomes and Survival in Patients with Severe COVID-19 in the Largest Health Care System in Central Florida. *medRxiv*, 2020.2008.2025.20181909 (2020).
13. Pritchard, M., *et al.* ISARIC Clinical Data Report 4 October 2020. *medRxiv*, 2020.2007.2017.20155218 (2020).
14. Intensive Care National Audit And Research Centre (ICNARC). ICNARC report on COVID-19 in criƟcal care:England, Wales and Northern Ireland (2020).
15. Hall, V., *et al.* Do antibody positive healthcare workers have lower SARS-CoV-2 infection rates than antibody negative healthcare workers? Large multi-centre prospective cohort study (the SIREN study), England: June to November 2020. *medRxiv*, 2021.2001.2013.21249642 (2021).
16. Dan, J.M., *et al.* Immunological memory to SARS-CoV-2 assessed for up to 8 months after infection. *Science* **371**, eabf4063 (2021).
17. To, K.K., *et al.* COVID-19 re-infection by a phylogenetically distinct SARS-coronavirus-2 strain confirmed by whole genome sequencing. *Clinical infectious diseases : an official publication of the Infectious Diseases Society of America* (2020).
18. Brazeau, N.F., *et al.* Report 34: COVID-19 Infection Fatality Ratio: Estimates from Seroprevalence. in *COVID-19 reports* (Imperial College London, 2020).
19. Salje, H., *et al.* Estimating the burden of SARS-CoV-2 in France. *Science* **369**, 208-211 (2020).
20. Polack, F.P., *et al.* Safety and Efficacy of the BNT162b2 mRNA Covid-19 Vaccine. *New England Journal of Medicine* **383**, 2603-2615 (2020).

21. Voysey, M., *et al.* Safety and efficacy of the ChAdOx1 nCoV-19 vaccine (AZD1222) against SARS-CoV-2: an interim analysis of four randomised controlled trials in Brazil, South Africa, and the UK. *The Lancet* **397**, 99-111 (2021).
22. Public Health England. PHE monitoring of the early impact and effectiveness of COVID-19 vaccination in England. 1-15 (Public Health England 2021).
23. Bernal, J.L., *et al.* Early effectiveness of COVID-19 vaccination with BNT162b2 mRNA vaccine and ChAdOx1 adenovirus vector vaccine on symptomatic disease, hospitalisations and mortality in older adults in England. *medRxiv*, 2021.2003.2001.21252652 (2021).
24. Jackson, L.A., *et al.* An mRNA Vaccine against SARS-CoV-2 - Preliminary Report. *N Engl J Med* **383**, 1920-1931 (2020).
25. Zhu, F.C., *et al.* Safety, tolerability, and immunogenicity of a recombinant adenovirus type-5 vectored COVID-19 vaccine: a dose-escalation, open-label, non-randomised, first-in-human trial. *Lancet (London, England)* **395**, 1845-1854 (2020).
26. Mulligan, M.J., *et al.* Phase I/II study of COVID-19 RNA vaccine BNT162b1 in adults. *Nature* **586**, 589-593 (2020).
27. Sahin, U., *et al.* COVID-19 vaccine BNT162b1 elicits human antibody and TH1 T cell responses. *Nature* **586**, 594-599 (2020).
28. Folegatti, P.M., *et al.* Safety and immunogenicity of a candidate Middle East respiratory syndrome coronavirus viral-vectored vaccine: a dose-escalation, open-label, non-randomised, uncontrolled, phase 1 trial. *The Lancet Infectious Diseases* **20**, 816-826 (2020).
29. Baden, L.R., *et al.* Efficacy and Safety of the mRNA-1273 SARS-CoV-2 Vaccine. *New England Journal of Medicine* **384**, 403-416 (2020).
30. Logunov, D.Y., *et al.* Safety and efficacy of an rAd26 and rAd5 vector-based heterologous prime-boost COVID-19 vaccine: an interim analysis of a randomised controlled phase 3 trial in Russia. *The Lancet* (2021).
31. Jones, S.P., Imperial College London Big Data Analytical Unit & YouGov Plc. Imperial College London YouGov Covid Data Hub. (YouGov Plc, 2021).
