# Supplementary material for: Modelling the impact of vaccine hesitancy in prolonging the need for Non-Pharmaceutical Interventions to control the COVID-19 pandemic
Source: Commun Med (Lond). 2022 Feb 10;2:14. doi: 10.1038/s43856-022-00075-x (PMC9053271; doi:10.1038/s43856-022-00075-x)
Supplement: Supplementary file 4 — Reporting Summary [file 43856_2022_75_MOESM4_ESM.pdf]

nature research

Corresponding author(s): Daniela Olivera Mesa

Last updated by author(s): Jan 17, 2022

Reporting Summary

Nature Research wishes to improve the reproducibility of the work that we publish. This form provides structure for consistency and transparency in reporting. For further information on Nature Research policies, see our Editorial Policies and the Editorial Policy Checklist.

Statistics

For all statistical analyses, confirm that the following items are present in the figure legend, table legend, main text, or Methods section.

n/a	Confirmed	
☐	☒	The exact sample size (*n*) for each experimental group/condition, given as a discrete number and unit of measurement
☒	☐	A statement on whether measurements were taken from distinct samples or whether the same sample was measured repeatedly
☒	☐	The statistical test(s) used AND whether they are one- or two-sided<br>*Only common tests should be described solely by name; describe more complex techniques in the Methods section.*
☐	☒	A description of all covariates tested
☐	☒	A description of any assumptions or corrections, such as tests of normality and adjustment for multiple comparisons
☐	☒	A full description of the statistical parameters including central tendency (e.g. means) or other basic estimates (e.g. regression coefficient) AND variation (e.g. standard deviation) or associated estimates of uncertainty (e.g. confidence intervals)
☒	☐	For null hypothesis testing, the test statistic (e.g. *F*, *t*, *r*) with confidence intervals, effect sizes, degrees of freedom and *P* value noted<br>*Give P values as exact values whenever suitable.*
☒	☐	For Bayesian analysis, information on the choice of priors and Markov chain Monte Carlo settings
☒	☐	For hierarchical and complex designs, identification of the appropriate level for tests and full reporting of outcomes
☒	☐	Estimates of effect sizes (e.g. Cohen's *d*, Pearson's *r*), indicating how they were calculated

Our web collection on statistics for biologists contains articles on many of the points above.

Software and code

Policy information about availability of computer code

Data collection	The COVID-19 vaccination model was written in R using the NIMUE package (https://github.com/mrc-ide/nimue). All code used for analyses is available on GitHub (https://github.com/mrc-321 ide/covid_vaccine_hesitancy or https://doi.org/10.5281/zenodo.5818075)
Data analysis	Data were analysed in R (version 4.0.2)

For manuscripts utilizing custom algorithms or software that are central to the research but not yet described in published literature, software must be made available to editors and reviewers. We strongly encourage code deposition in a community repository (e.g. GitHub). See the Nature Research guidelines for submitting code & software for further information.

Data

Policy information about availability of data

All manuscripts must include a data availability statement. This statement should provide the following information, where applicable:

- Accession codes, unique identifiers, or web links for publicly available datasets
- A list of figures that have associated raw data
- A description of any restrictions on data availability

All data used in this study are from publicly available sources at the links provided in the main text and references. Vaccine hesitancy surveys are from the Imperial College London YouGov Covid 19 Behaviour Tracker Data Hub (https://github.com/YouGov-Data/covid-19-tracker). For ease of reproducibility of our results, the dataset is also stored in our associated publicly available Github repository (https://doi.org/10.5281/zenodo.5818075). Demographic information is from the United Nations Population prospects https://population.un.org/wpp/). Mobility data from Google (https://www.google.com/covid19/mobility). And model fittings to country-specific data are from https://mrc-ide.github.io/global-lmic-reports/results. Source data can be found in the Supplementary Data file

Field-specific reporting

Please select the one below that is the best fit for your research. If you are not sure, read the appropriate sections before making your selection.

☒ Life sciences ☐ Behavioural & social sciences ☐ Ecological, evolutionary & environmental sciences

For a reference copy of the document with all sections, see nature.com/documents/nr-reporting-summary-flat.pdf

Life sciences study design

All studies must disclose on these points even when the disclosure is negative.

Sample size	The manuscript does not report primary data collection but shows simulated results of COVID-19. The model has been parameterized using publicly available data which are reported in full elsewhere.
Data exclusions	No data were excluded from this analysis.
Replication	A deterministic model was implemented to simulate the results. Therefore, there were not replicate simulations consistent with this type of models. However, to capture survey uncertainty, answers per age group were used to parameterise a multinomial distribution, from which we drew 100 replicates. For each replicate a deterministic model was run. Median, 10% and 90% quantiles are presented in results
Randomization	There was no randomization in this analysis consistent with modeling studies of this type.
Blinding	There was no blinding in these analyses consistent with modeling studies of this type.

Reporting for specific materials, systems and methods

We require information from authors about some types of materials, experimental systems and methods used in many studies. Here, indicate whether each material, system or method listed is relevant to your study. If you are not sure if a list item applies to your research, read the appropriate section before selecting a response.

Materials & experimental systems

n/a	Involved in the study	
☒	☐	Antibodies
☒	☐	Eukaryotic cell lines
☒	☐	Palaeontology and archaeology
☒	☐	Animals and other organisms
☒	☐	Human research participants
☒	☐	Clinical data
☒	☐	Dual use research of concern

Methods

n/a	Involved in the study	
☒	☐	ChIP-seq
☒	☐	Flow cytometry
☒	☐	MRI-based neuroimaging
