# Supplementary material for: Modelling the impact of vaccine hesitancy in prolonging the need for Non-Pharmaceutical Interventions to control the COVID-19 pandemic
Source: Commun Med (Lond). 2022 Feb 10;2:14. doi: 10.1038/s43856-022-00075-x (PMC9053271; doi:10.1038/s43856-022-00075-x)
Supplement: Supplementary file 5 — Description of Additional Supplementary Files [file 43856_2022_75_MOESM5_ESM.pdf]

Description of Additional Supplementary Files

File Name: Supplementary Data

Description: Source data for projected COVID-19 dynamics given vaccine hesitancy (Figure 1); cumulative deaths and hospitalisations for the vaccinated and unvaccinated populations at the end of the projection horizon (Figure 2); stringency of non-pharmaceutical interventions required to control the epidemic under different vaccine hesitancy scenarios (Figure 3) ; and impact of vaccine hesitancy for France, Germany and U.K (Figure 4)
